# Two gene clusters are required for mannosylerythritol lipid biosynthesis in *Sporisorium reilianum*

**DOI:** 10.1128/mbio.00899-25

**Published:** 2025-08-18

**Authors:** Jessica Tiefenbacher, Uwe Linne, Johannes Freitag, Björn Sandrock

**Affiliations:** 1 Department of Biology, Philipps-University Marburg 9377, Marburg, Germany; 2 Department of Chemistry, Philipps-University Marburg 9377, Marburg, Germany; Cornell University, Ithaca, New York, USA

**Keywords:** *Sporisorium reilianum*, MEL biosynthesis, biosynthetic gene clusters, surface tension, emulsification

## Abstract

Mannosylerythritol lipids (MELs) are antibacterial surface-active molecules produced by several basidiomycetous fungi. Enzymes for MEL biosynthesis are encoded in a gene cluster composed of five genes, which are both necessary and sufficient for the biosynthesis of MELs. Here, we report that in two closely related biotrophic pathogens*—Sporisorium reilianum f. sp. reilianum* (*SrS*) and *Sporisorium reilianum SRZ2*—MEL production is achieved through the combined activity of two gene clusters. While the larger cluster is required for production of the disaccharide moiety, for attachment of one acyl chain to the sugar backbone and for export of MELs, the smaller cluster encodes two enzymes for additional acylation of the sugar backbone and is essential to produce mature MELs in *S. reilianum*. The genes in the smaller cluster appear to be duplications of two of the original genes in the larger cluster, which are not expressed in *SrS* but in related fungi, e.g., *Ustilago maydis*. MELs made by *SrS* are tri-acylated and exhibit altered physical and chemical properties, making them interesting candidates for future biotechnological and pharmaceutical applications. In summary, our study showcases how duplication and diversification of MEL clusters can lead to the evolution of novel biosurfactants.

**IMPORTANCE:**

Secondary metabolites produced by fungi can act as weapons against competitors, can help access nutrients, or assist development and communication. One group of secondary metabolites are surface-active glycolipids that have a great potential as biodegradable detergents. Upon nitrogen starvation, biosynthesis of the glycolipid mannosylerythritol lipid (MEL) is induced by a gene cluster composed of five genes in the plant pathogen *Ustilago maydis* and related basidiomycetous fungi. This study shows that in the smut fungus *Sporisorium reilianum f. sp. reilianum,* the five genes of the MEL gene cluster were not sufficient to produce the isolated MEL variant. In contrast to conventional MELs, MELs of *S. reilianum* are tri-acylated at the mannose moiety with acyl groups in the range of C_6_–C_10_. These MELs exhibit altered physical and chemical properties, making them interesting novel candidates for future applications. Furthermore, we demonstrate that in *S. reilianum,* a combination of two gene clusters is necessary for MEL biosynthesis, which enables a glimpse into the evolutionary history of the altered MEL species.

## INTRODUCTION

**Fig 1:**
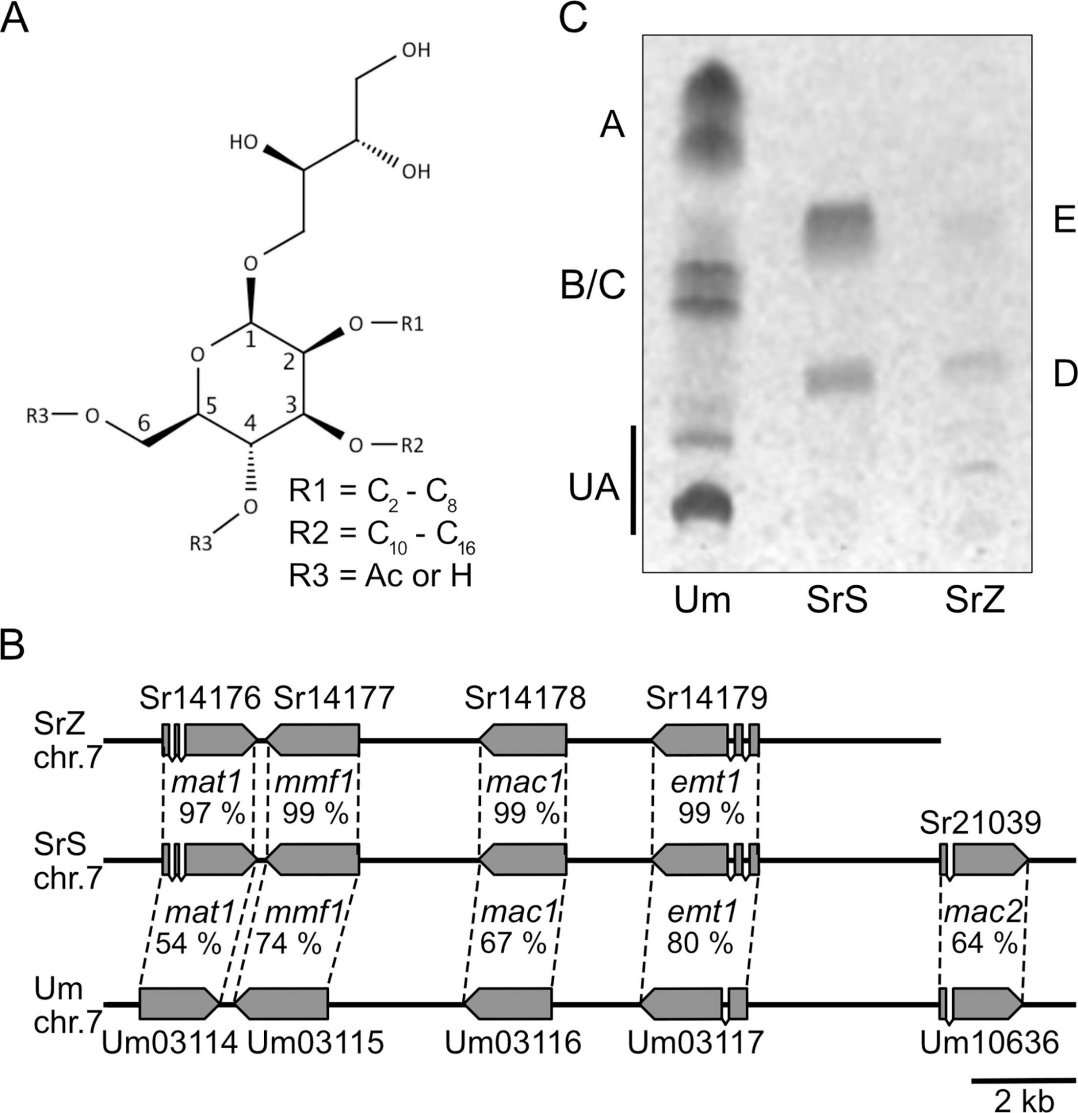
MEL synthesis in *Sporisorium reilianum*. (**A**) Structure of *U. maydis* MEL with MEL-A: R3 = Ac; MEL-B: R3 at C6 = Ac, R3 at C4 = H; MEL-C: R3 at C6 = H, R3 at C4 = Ac; MEL-D: R3 = H. Ac = acetyl group. (**B**) Alignment of MEL gene clusters from *U. maydis* (*Um*), *S. reilianum f. sp. reilianum* (*SrS*), and *S. reilianum SRZ2* (*SrZ*). The protein identity is given in %. (**C**) TLC of MELs synthesized by the different fungi. MEL-E is the novel tri-acylated variant. UA: ustilagic acid.

Identification and characterization of novel microbial-derived biosurfactants with the potential to replace oil-based surfactants are crucial for the development of biodegradable detergents. Several of these biosurfactants are already produced in industrial scales; however, they are still less cost-efficient compared to their synthetic alternatives (1–4). One group of biosurfactants is surface-active glycolipids such as rhamnolipids, sophorolipids, and mannosylerythritol lipids (MELs) (Fig. 1A) and cellulose lipids, which all contain distinct sugar moieties (5). MELs have interesting properties, including the reduction of surface tension, emulsification of organic compounds, and antibiotic activity against gram-positive bacteria (6–8).

MELs are synthesized and secreted by a group of basidiomycetous fungi in the clade of *Ustilaginaceae*. MEL production is induced upon nitrogen starvation conditions by a gene cluster composed of five genes (9) (Fig. 1B). In the first step, Emt1 connects an erythritol moiety with a mannose molecule at the C1 position (10). This compound is then decorated with fatty acid side chains inside of vesicular organelles termed peroxisomes through the activity of acyltransferases Mac1 and Mac2 at the C2 and C3 residues of the mannosyl part (Fig. 1A) (9, 11, 12). This directly couples MEL biosynthesis to fatty acid oxidation occurring in this organelle (11, 13, 14). Acetylation at the mannose residues C4 and/or C6 by Mat1 occurs at the plasma membrane and is necessary for active transport through the transporter of the major facilitator family Mmf1 (9, 15). Depending on the acetylation state, MELs can be classified into MEL-A, MEL-B, MEL-C, and MEL-D. MEL-A is di-acetylated, and MEL-D is non-acetylated. Mono-acetylated variants are acetylated at the C4 position (MEL-C) or the C6 position (MEL-B).

*Sporisorium reilianum f. sp. reilianum* is a basidiomycetous fungus infecting sorghum (*SrS*) and is closely related to *Sporisorium reilianum SRZ2* (*SrZ*), which infects maize plants (16–18). Whether both species produce MELs was unknown so far. Therefore, we investigated the biosynthesis of MELs in *SrS* and *SrZ*. We found that both fungi produce unusual MEL variants. For *SrS,* we provide a detailed genetic analysis, which reveals that MELs emerge from the activity of two gene clusters in this species.

## RESULTS

### *S. reilianum* produces MELs distinct from *U. maydis*

In the genomes of both sequenced varieties of *S. reilianum* (*SrZ* and *SrS*), a putative gene cluster for MEL biosynthesis was identified by homology-based searches using the National Center for Biotechnology Information (NCBI) databases search programs BLASTp and BLASTn. While *SrS* contains homologs of all five genes that occur in the *U. maydis* gene cluster, no homolog of *mac2* was encoded in the *SrZ* genome (Fig. 1B). The protein sequences from both *S. reilianum* varieties exhibit very high similarities (Fig. 1B). Nitrogen limitation was known to induce glycolipid expression in *U. maydis* (19), and therefore we also used this condition to test glycolipid production by the *Sporisorium* species. We observed biosynthesis of MELs by *SrZ* and by *SrS*. The migration pattern of MEL variants differed remarkably from that of MELs produced by *U. maydis* when analyzed by thin layer chromatography (TLC) (Fig. 1C). Two bands appeared for *SrS* and *SrZ,* migrating slower in thin-layer chromatography (TLC) experiments when compared to MEL-A and MEL-B/C species of *U. maydis* (Fig. 1C). This suggests an overall lower hydrophobicity of *S. reilianum-*derived MEL molecules.

**Fig 2:**
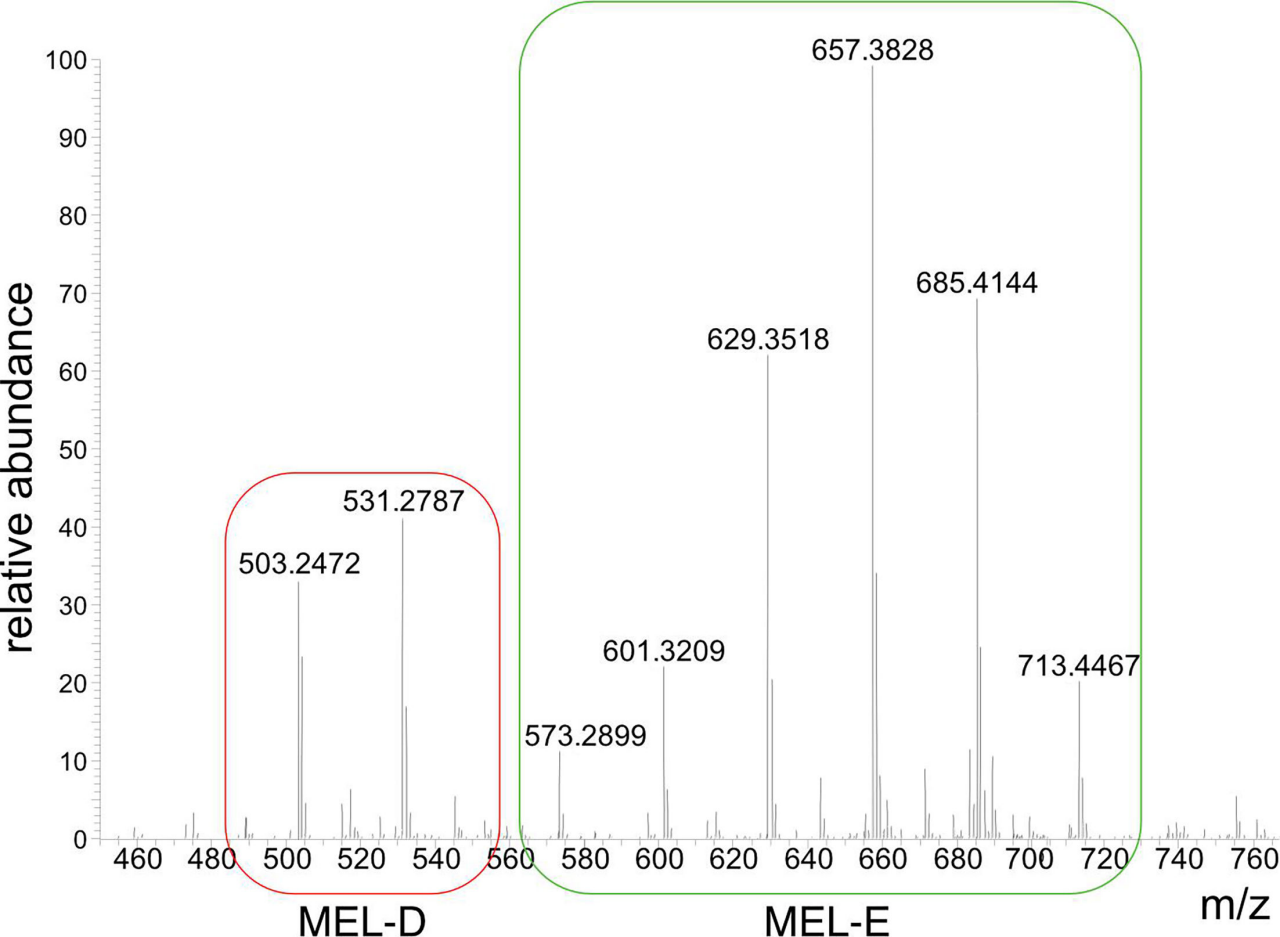
LCMS analysis of MELs from *SrS*. Averaged mass spectra of MELs from *SrS* analyzed by LCMS. Mass fragmentation (MS^2^) revealed two main masses for MEL-D variants (red area) and six main masses for MEL-E (green area).

These data were already surprising, especially because *SrZ* does not contain a *mac2* gene, which has been shown to be critical for MEL production in *U. maydis* (9). Due to the larger amounts of MELs, we focused on *SrS* and analyzed the exact MEL composition by high-performance liquid chromatography coupled with mass spectrometry (HPLC/MS). Two prominent masses for the slower migrating MEL variants (MEL-D) and six for the faster migrating MEL variants were observed (Fig. 2; Table 1). The faster migrating MELs are tri-acylated MEL variants with mostly C_6_ and C_8_ fatty acid side chains (Table 1). Comparing the spectra with the mass 629.35 m/z from *Um* and *SrS*, no acetylation was detected in MS^2^ analysis of *SrS* mass fragmentation (Fig. S1A and B), and thus we will refer to these novel species as MEL-E (11).

**TABLE 1:** Fatty acid composition of *SrS* MEL variants at mannose residues C2, C3, and either C4 or C6 derived from MS^2^ data

*m/z*	Fatty acid composition
MEL-D	
503.2472	C_6_, C_6_
531.2787	C_6_, C_8_
MEL-E	
573.2899	C_2_, C_8_, C_6_
601.3209	C_6_, C_6_, C_6_
629.3518	C_6_, C_8_, C_6_
657.3828	C_6_, C_8_, C_8_
685.4144	C_8_, C_8_, C_8_
	C_6_, C_10_, C_8_
713.4467	C_8_, C_10_, C_8_
	C_6_, C_10_, C_10_

### Mac2 and Mat1 are not required for MEL production in *SrS*

**Fig 3:**
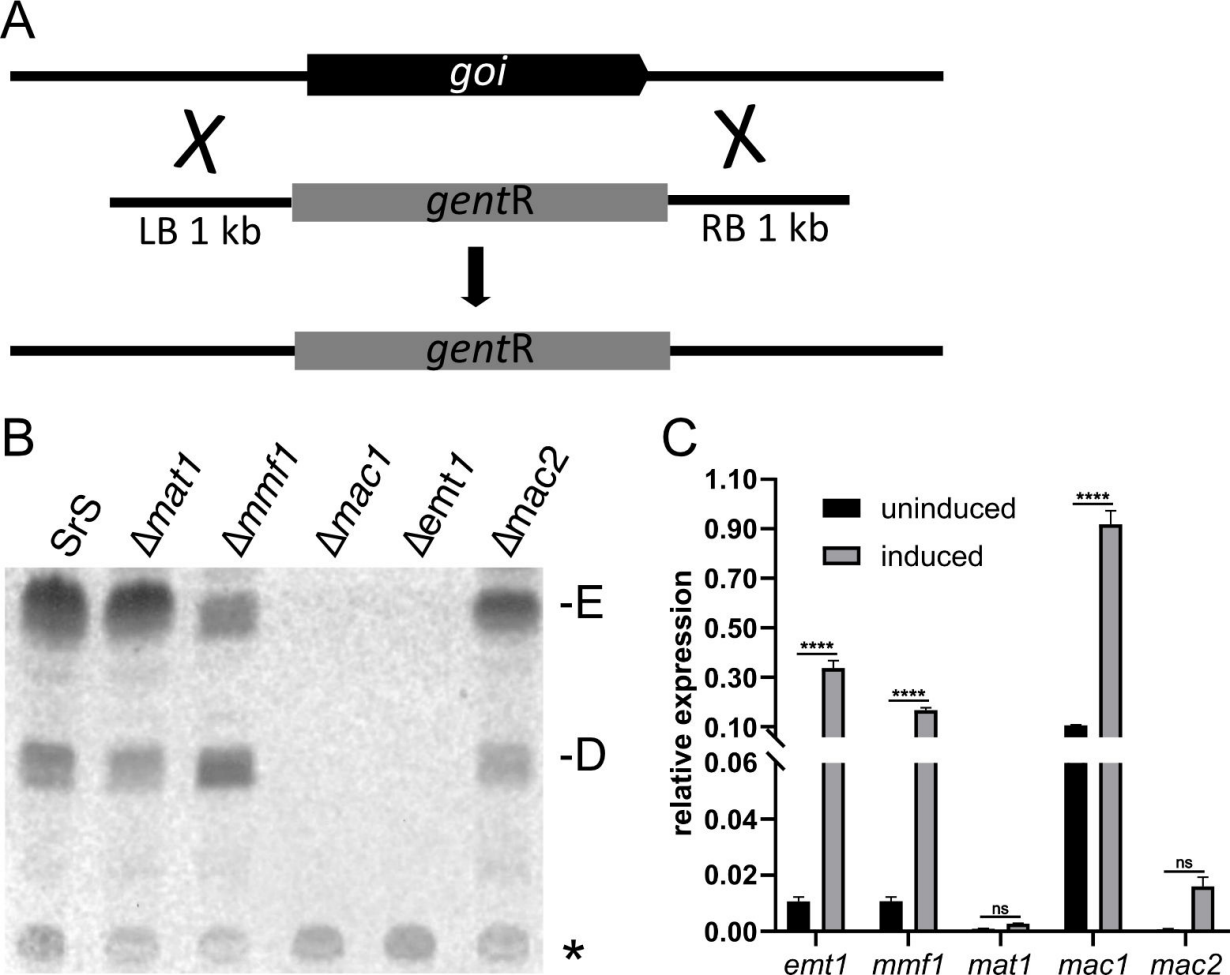
Mat1 and Mac2 are obsolete for MEL production in *SrS*. (**A**) Knock-out strategy using PCR-based amplification of 1 kb left (LB) and right border (RB) of the gene of interest (goi). (**B**) TLC of MELs synthesized by the individual *SrS* ko-strains. D, MEL-D; E, MEL-E. The asterisk denotes an unknown compound not related to the MEL gene cluster under investigation. (**C**) qPCR data for expression of all the five MEL genes of cells grown in the presence of nitrogen (uninduced) or of cells incubated for 6 h in nitrogen starvation conditions (induced). Each bar represents the mean of three experiments. Error bars represent standard deviation. The significance of the results was analyzed with two-way ANOVA: ****: *P* < 0.001; ns: non-significant.

To characterize MEL production in *Sporisorium* species in more detail, we constructed deletion mutants of all five genes of the MEL cluster in *SrS* via homologous recombination using the geneticin resistance cassette with 1 kb flanking regions to replace the individual genes (Fig. 3A) (20). SrEmt1 and SrMac1 were required for MEL production in accordance with what is known from *U. maydis*. The *mmf1* deletion strain produced similar amounts of MEL-E and MEL-D. These phenotypes could be rescued by reintegration of the corresponding genes (Fig. S2).

However, deletion of *mac2* and *mat1* did not interfere with MEL biosynthesis at all (Fig. 3B). These results, together with the previous notion that *SrZ* does not contain a gene encoding Mac2, suggest a so-far uncharacterized way to achieve MEL biosynthesis.

Next, we analyzed the expression of the mRNAs of the MEL genes under nitrogen starvation conditions by qPCR (Fig. 3C). Expression of all five genes was detected upon nitrogen starvation, but to a very different extent. While mRNAs of *mmf1*, *mac1,* and *emt1* were highly induced, the relative expression levels for *mat1* and *mac2* were very low. This is in accordance with our results obtained with the deletion mutants. *SrS_mat1* and *SrS_mac2* are not involved in the biosynthesis of MELs in *SrS* (Fig. 3B and C).

To examine whether the open reading frames encode functional Mat1 and Mac2 in *SrS*, we used heterologous complementation experiments in *U. maydis* (Fig. S3). Constitutive expression of each gene of the *SrS* MEL cluster was able to rescue the respective *U. maydis* deletion mutant (Fig. S3A and B) (9, 10, 15). Analysis of LC/MS and MS^2^ data are summarized in the heat maps (Fig. S3C), indicating that SrMac1 incorporates C_6_ and C_8_ fatty acids at R1 and SrMac2 C_10_, C_12_ and C_14_ fatty acids at R2. Expression of SrMat1 triggered formation of MEL-B/C in *U. maydis* via acetylation at either C4 or C6 of the mannose moiety (Fig. S4). The results obtained for SrMat1 (acetylation) and SrMac2 (acylation using C_10_–C_14_ fatty acids) show that SrMat1 and SrMac2 are *per se* functional, but not expressed in *SrS* under nitrogen starvation conditions.

### A second gene cluster participates in MEL production in *SrS*

**Fig 4:**
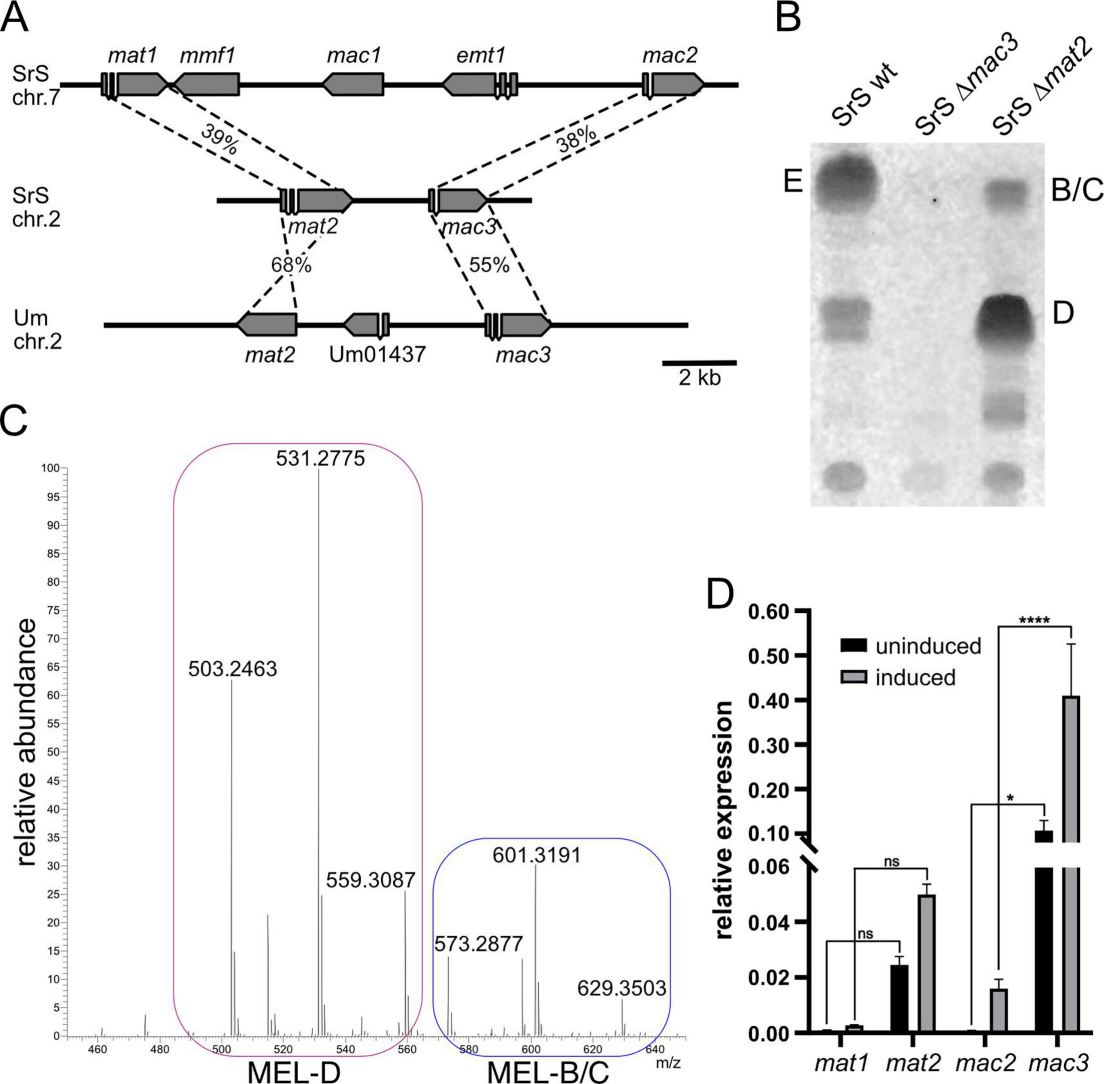
A novel gene cluster is required for MEL biosynthesis in *S. reilianum*. (**A**) Scheme to illustrate the relation of the novel gene clusters of *U. maydis* and *SrS* with the larger MEL cluster of *SrS*. (**B**) TLC of MELs synthesized by the indicated *SrS* deletion strains. (**C**) Averaged mass spectra of MELs from *SrS ∆mat2* analyzed by LC-MS. Mass fragmentation (MS^2^) revealed three main masses for MEL-D variants (red area) and three main masses for MEL-B/C (blue area). (**D**) qPCR data for expression of *mat2* and *mac3* genes of cells grown in the presence of nitrogen (uninduced) or of cells incubated for 6 h in nitrogen starvation conditions (induced). Each bar represents the mean of three experiments. Error bars represent standard deviation. The significance of the results was analyzed with two-way ANOVA: *: *P* < 0.05; ****: *P* < 0.001; ns: non-significant.

MELs from *SrS* contain fatty acid side chains of C_6_, C_8_, or C_10_ at C3 of the mannosyl moiety in the sugar backbone. Previously, we have described Mac3 of *U. maydis*, which was able to replace UmMac2 in the MEL biosynthesis (21, 22). UmMac3 is able to add such shorter fatty acids (C_6_, C_8_, or C_10_) to the sugar (21). The corresponding gene is located on chromosome 2 together with a gene for a putative homolog of UmMat1 (9). Due to the similarities of fatty acids incorporated in MELs of *SrS* and the substrate range of UmMac3, we hypothesized that a Mac3-containing gene cluster might be involved. Thus, we searched for a *mac3* gene in the genome of *SrS* via BLASTp. Indeed, similar to *U. maydis, SrS*_*mac3* is located on chromosome 2 next to a putative *SrS*_*mat2* gene constituting a second syntenic gene cluster (Fig. 4A).

To test whether this cluster is essential in *SrS*, we deleted both genes individually via homologous recombination. Subsequently, MEL production was analyzed (Fig. 4B). The deletion of *mac3* in *SrS* resulted in a complete loss of MEL production, revealing its essential role for the MEL biosynthesis pathway. Mass spectrometric analysis of MELs produced by ∆*mat2* cells showed that almost no MEL-E species were synthesized (Fig. 4C; Table 2). Analysis of gene expressions of *mac3* and *mat2* by qPCR demonstrated that both genes are induced upon nitrogen starvation, unlike *mat1* and *mac2* (Fig. 4D).

**TABLE 2:** Fatty acid composition of *SrS ∆mat2* MEL variants at mannose residues C2, C3, and either C4 or C6 derived from MS^2^ data

*m/z*	Fatty acid composition
MEL-D	
503.2463	C_6_, C_6_
531.2775	C_6_, C_8_
559.3087	C_8_, C_8_
MEL-B/C	
573.2877	C_2_, C_8_, C_6_
601.3191	C_2_, C_8_, C_8_
	C_2_, C_10_, C_6_
629.3503	C_2_, C_10_, C_8_

Next, we complemented the deletion strains *SrS*∆*mac3* and *SrS*∆*mat2* with the corresponding genes (Fig. S5A and B). Expression of both SrMac3 and SrMat2 rescued the phenotypes of respective deletion mutants. In addition, we expressed UmMac2 and SrMac2 in *SrS∆mac3* strains (Fig. S5B). Expression of both proteins could restore MEL synthesis (Fig. S5A). However, few MELs were produced, and the acyl groups incorporated by both Mac2 versions were restricted to C_10_ and C_12_ (Fig. S5C). In *U. maydis,* UmMac2 adds fatty acids up to a length of C_16_, but neither C_14_ nor C_16_ side chains were detected upon analysis of the LC/MS fragmentation patterns (9, 12). Substrate availability in the peroxisomes of *SrS* is one potential explanation for this difference.

### Characterization of the physical and biological features of *S. reilianum* MELs

**Fig 5:**
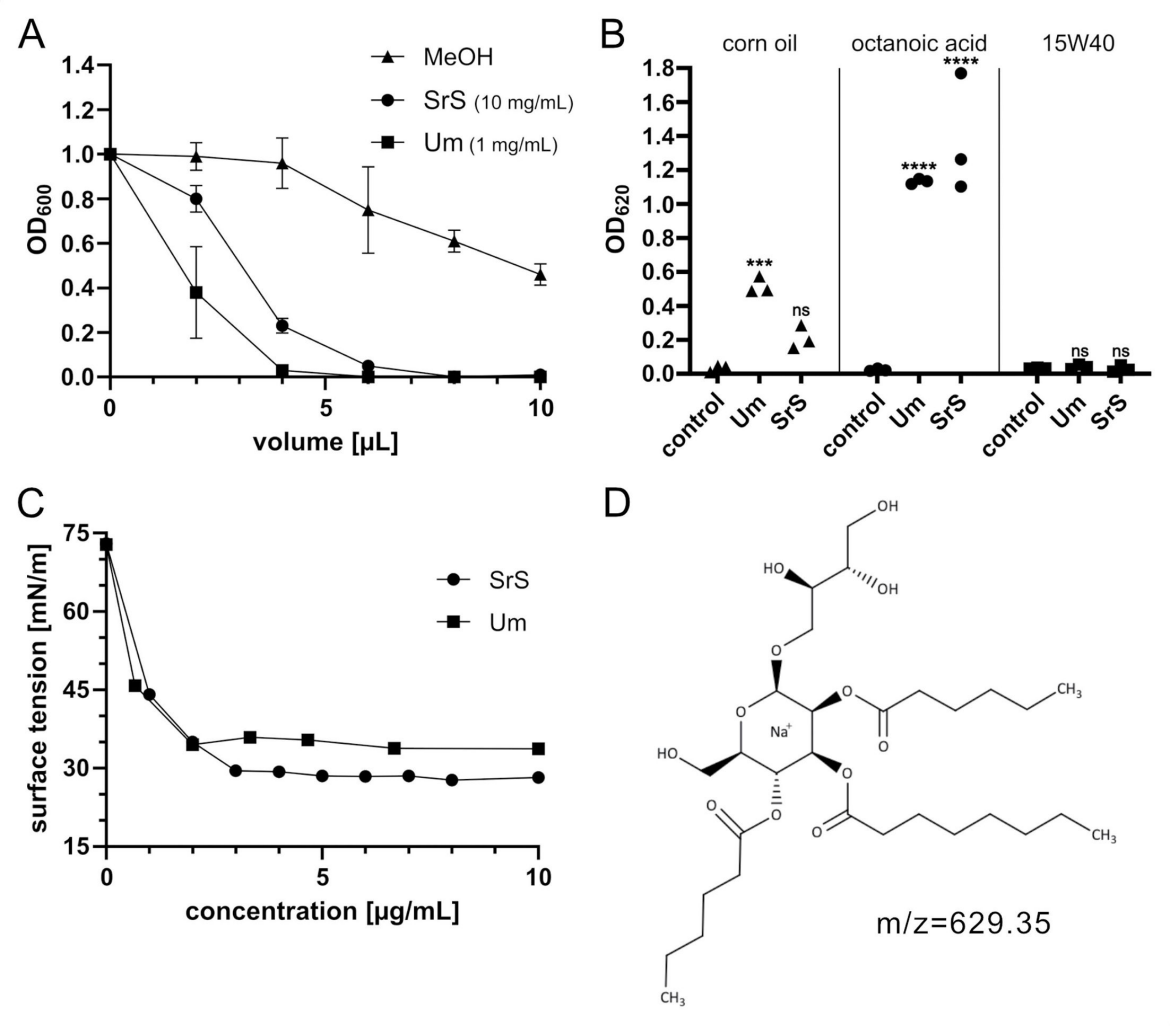
Characteristics of *S. reilianum* MELs compared to *U. maydis* MELs. (**A**) Activity of MELs against *Bacillus subtilis* after 13 h growth. Mean values together with the SD are shown for three independent experiments. (**B**) Emulsification of different fatty acid-containing substances in water in the absence or presence of MELs. Each symbol represents one single experiment. The significance of the results was analyzed with two-way ANOVA: ****: *P* < 0.001; ns: non-significant. (**C**) Surface tension analysis of MELs in water. (**D**) Tri-acylated MEL structure. The C_6_ fatty acid might be either at C4 (shown here) or at C6 of the mannose.

To further characterize MELs from *S. reilianum,* we investigated several of their properties in comparison to *U. maydis* MELs. First, we compared the activity against *Bacillus subtilis*, a gram-positive bacterium affected by standard MELs from *U. maydis* (21). *Um* MELs were collected from MB215 ∆*rua1*. This strain is unable to produce ustilagic acids, a second class of glycolipids produced by *U. maydis* and thus, allows us to specifically analyze MELs (23). *SrS* MELs inhibited growth of *B. subtilis* with an LD50 of 0.32 mg/mL (Fig. 5A). This is approximately 20-fold less efficient than *Um* MELs (MEL-A/B/C) with an LD50 of 0.016 mg/mL or the non-acetylated *Um* MELs (MEL-D) (21).

Next, we have measured the emulsification of three different organic substances: corn oil (C_16_ and C_18_), octanoic acid (C_8_), and 15W40 motor oil (C_20_-C_24_) in water and in the absence or presence of 20 µg MELs (Fig. 5B). *SrS* MELs were efficient in the emulsification of octanoic acid but did not show a strong effect on corn or motor oil. *Um* MELs were able to emulsify corn oil and octanoic acid but showed no activity on motor oil. These data suggest that the emulsification ability likely relies on the length of the acyl groups attached to the sugar backbone. MELs with longer side chains appear to emulsify longer hydrophobic substances.

Finally, we determined how *SrS* MELs affected the surface tension of water using the Wilhelmi method (Fig. 5C) and compared this to *U. maydis* MELs. We could show that both substances significantly reduced the surface tension of water, hence acting as biosurfactants. MELs of *S. reilianum* appeared even slightly more effective.

## DISCUSSION

In our study, we have demonstrated that the plant pathogenic fungus *S. reilianum* synthesizes a novel MEL variant in which the mannose residue contains three acyl groups (Fig. 5D). The MELs (MEL-A, -B, and -C) described for other species are di-acylated at C2 and C3 and di- or mono-acetylated at C4 and/or C6 of the mannose (Fig. 1A). The previously identified tri-acylated MELs synthesized by *Pseudozyma* species resemble MEL-A molecules additionally acylated at the erythritol moiety by longer fatty acids C_16_–C_18_ (24–26). They exhibit a higher hydrophobicity in TLC experiments compared to MEL-A and are distinct from the tri-acylated MELs we have identified (Fig. 1B) (25). The newly identified tri-acylated MELs lack any acetylation at C4 and C6 of the mannose but contain one short-chain fatty acid (C_6_ or C_8_) instead. Therefore, they represent a novel class of MELs, termed MEL-E.

**Fig 6:**
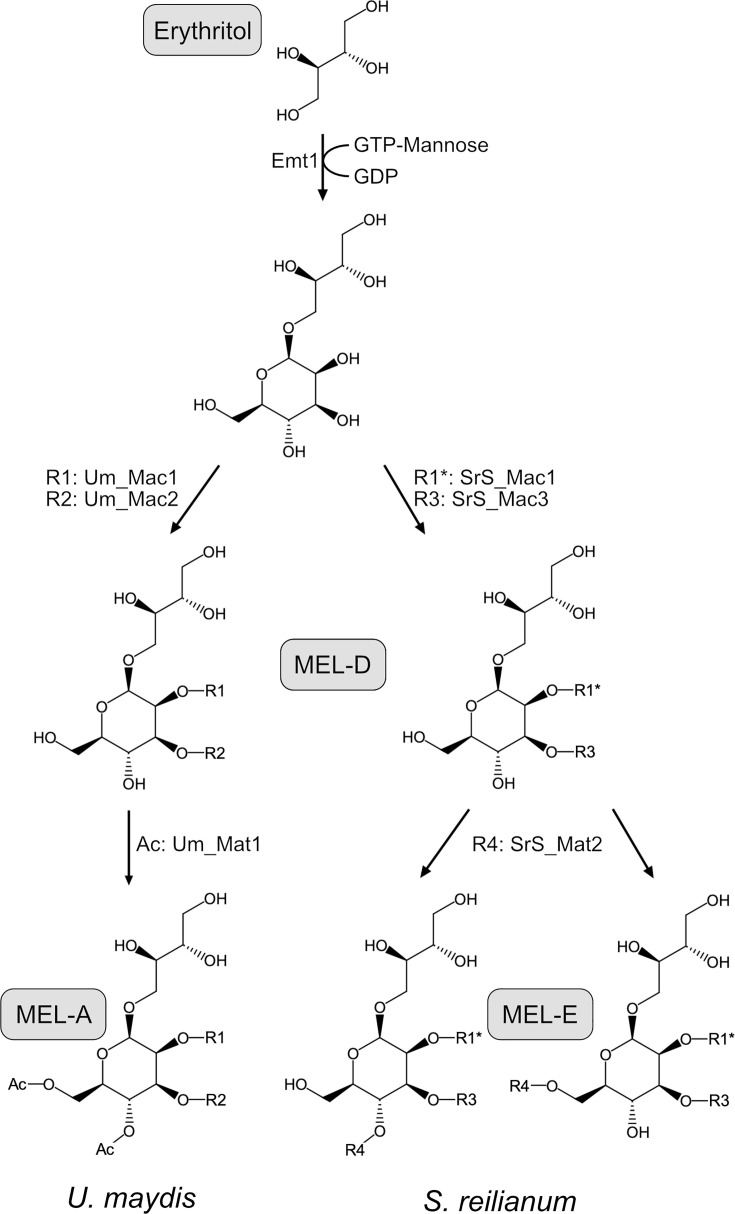
Biosynthesis pathway for MELs in either *U. maydis* or *S. reilianum*. R1: C_2_, C_4_, and C_6_ fatty acids; R1*: C_6_ and C_8_ fatty acids; R2: C_12_, C_14_, and C_16_ fatty acids; R3: C_6_, C_8_, and C_10_ fatty acids; R4: C_6_ and C_8_ fatty acids. Ac = acetyl group (C_2_ fatty acid).

We have observed that two genes (*SrS*_*mat1* and *SrS*_*mac2*) of the classical MEL gene cluster remained silent even under conditions where MELs are normally produced. However, the genomes of *SrS* and *SrZ* contain a second gene cluster encoding *SrS*_*mac3* and *SrS*_*mat2*. We could show that SrMac3 replaces SrMac2 for the acylation of C3 and that SrMat2 took over the role of SrMat1 adding one short-chain fatty acid (C_6_ or C_8_) at the C4 or the C6 residue of the mannose (Fig. 6). MEL biosynthesis via the conventional MEL pathway (*U. maydis*) relies on the five genes of the larger gene cluster. The newly identified MEL pathway (*S. reilianum f. sp. reilianum*) combines two genes of the smaller gene cluster with three genes of the larger gene cluster (Fig. 6).

Although SrMat2 is not a real acetyltransferase like SrMat1 but rather a Mac enzyme, we have decided to keep the term Mat2 due to its sequence homology to the Mat1 proteins. Previously, we have demonstrated that in *U. maydis,* UmMac3 can complement a ∆*mac2* deletion strain, but is obsolete for the production of the majority of native MELs in this fungus (21). UmMac3 has a similar substrate range as SrMac3 (Table 1; 21). UmMat2 remains to be characterized.

**Fig 7:**
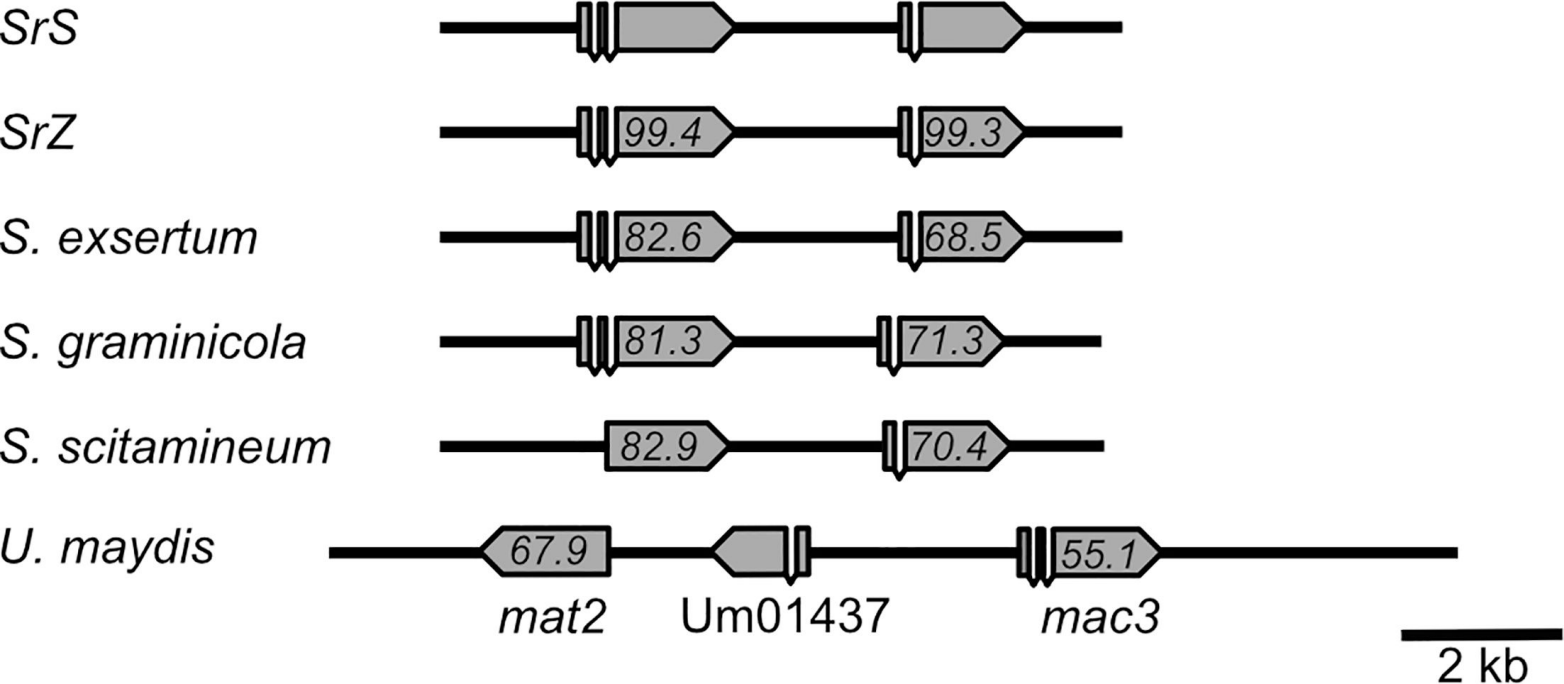
Conservation and synteny of the small gene clusters for MEL production. Syntenic gene clusters identified in the genomes of the indicated fungal organisms. For *S. exsertum,* the gene cluster has been identified and annotated using the genomic sequence provided by NCBI (BioProject PRJEB50360). The values are percent identities to the *SrS* Mat2 and Mac3 proteins.

The smaller gene cluster is unique to a few closely related fungi like *U. maydis*, *Sporisorium scitamineum*, *Sporisorium graminicola,* and *Sporisorium exsertum* (NCBI) (Fig. 7). While it appears to be mostly silent in *U. maydis,* it is co-regulated with the larger gene cluster for the production of tri-acylated MELs in *SrS*. We suggest that the smaller gene cluster is derived from a duplication in a common ancestor of this small group of smut fungi; after that, diversification took place. Phylogenetic analysis using BLASTp supports this duplication event (Fig. S6 to S9). For both proteins expressed from the small gene clusters, the closest relatives belong to the original large MEL cluster. Most probably, the smaller cluster only emerged once as it is always contained on chromosome 2 and shows high levels of synteny. In *U. maydis,* there occurred another rearrangement, which led to the engulfment of the gene *umag_01437,* whose orthologs are elsewhere on chromosome 2 in the *Sporisorium* species. While in *U. maydis,* MEL production relies mainly on the original cluster, the smaller cluster became essential in *S. reilianum*. It is unclear why the inactivation of Mac2 and Mat1 occurred in some of the species. One possible explanation for diversification of substrate specificity in Mac2 and Mac3 and subsequent selection on Mac3 via inactivation of Mac2 could be differences in the available substrate spectrum inside of peroxisomes. This idea is corroborated by our finding that UmMac2 can complement a deletion of *mac3* in *S. reilianum* (Fig. S5); however, many acyl groups are attached to the sugar backbone. On the contrary, SrMac2 uses longer substrates upon expression in *U. maydis,* but not upon expression in *S. reilianum*. Thus, it might indeed be the accessibility of β-oxidation intermediates in each of the fungi that determines the fate of *mac2* and *mac3* genes. For the substitution of *mat1* with *mat2,* this hypothesis is less plausible.

The biological function of MELs is still elusive. Also, why β-oxidation of fatty acids and the presence of peroxisomes are relevant for the infection by *U. maydis* has also not been fully understood (27, 28). The MEL gene cluster is upregulated on the plant surface prior to invasion of the host, a developmental stage coinciding with nitrogen starvation at least in the experimental setup routinely used (29). MEL biosynthesis is obsolete for infection by *U. maydis* under such experimental conditions (10). Interestingly, the shorter MEL cluster is restricted to a few plant pathogenic smut fungi and is even lost in the nonpathogenic species, e.g., *Pseudozyma hubeiensis SY62* (Fig. S6 and S7). To understand the function of MELs in particular in relation to the host, it will be useful to characterize *S. reilianum mac3* and *mat2* mutants in infection experiments as they showed different biophysical properties.

The characterization of the properties of novel variants has emerged as an important topic (30–32). Here, we found that *SrS* MELs inhibit growth of *B. subtilis* but to a lesser extent than *U. maydis* MELs. The mechanism of how the growth inhibition of gram-positive bacteria works is still unknown (33). The gram-negative bacterium *Escherichia coli* is not affected by MELs of *M. aphidis* and *U. maydis* (21). Studies with MEL-B against *Staphylococcus aureus* (gram-positive) have shown that the cell wall becomes decorated with the glycolipids, but this binding did not cause obvious structural alterations of bacterial cells (34). Moreover, we demonstrated that the critical micelle concentration (CMC) value of *SrS* MELs is in the same range or even lower than that of *Um* MELs. In a previous study, Morita et al. (35) have also determined the necessary force values for CMCs with MELs from other fungi like *Pseudozyma antarctica* (25.3 mN/m), *U. scitaminea* (25.6 mN/m), and *P. siamensis* (29.8 mN/m), all falling into the same range (35). This indicates that the tri-acylated MEL-E variant has a similar surface tension activity as the previously characterized MEL-A, -B, or -C. The emulsification activity of the *SrS* MELs is highest for octanoic acid. This may result from the major chain lengths of C_6_ and C_8_ or from the tri-acylation or from both. In a previous study, we have shown that non-acetylated tailor-made MEL-D with C_8_ or C_10_ at C2 and C_14_ and C_16_ at C3 was also a very effective emulsifier of octanoic acid (21). On the other hand, MEL-D with C_8_ or C_10_ at C2 and C_10_ and C_12_ at C3 was not able to emulsify octanoic acid. Thus, efficient emulsification either needs tri-acylated MEL-E with short-chain fatty acids (e.g., 3× C_8_) or di-acylated MEL-D with one short chain (e.g., C_8_) and one long-chain fatty acid (e.g., C_16_). We suggest that the newly identified tri-acylated MEL-E variants should be further explored as promising candidates for biodegradable biosurfactants.

## MATERIALS AND METHODS

### Strains and growth conditions

Fungal strains were grown in liquid YEPSL (1% yeast extract, 0.4% peptone, and 0.4% sucrose) or on solid potato dextrose broth containing 1.5% Bacto agar at 28°C and 23°C, respectively. The fungal strains used and generated in this study are listed in Table S1. *Sporisorium reilianum f. sp. zeae SRZCX112* (36) and *Sporisorium reilianum f. sp. reilianum SRS3* (H2)−8 (16) were kind gifts from Regine Kahmann (MPI Marburg, Germany). For the cross-species complementation experiments, the *U. maydis* strain MB215 (10) and its derivatives were used. To induce glycolipid production, *U. maydis* strains were grown to the stationary phase in YEPS-L and then transferred to the nitrogen starvation medium (OD_600_ = 0.1) containing 0.17% YNB (yeast nitrogen base without ammonia) and 1% glucose as carbon source. Glycolipid production in *S. reilianum* strains was induced analog using 2% glucose as the carbon source and, for ARS plasmid-containing strains only, 2 µg/mL Carboxin (Fluka). Glycolipid production was then allowed for 6 days at 23°C under rotation. *Escherichia coli* strain Top10 (Invitrogen) was used for transformation according to Hanahan et al. (37) and amplification of plasmid DNA (37). *Bacillus subtilis* wild-type strain was a kind gift from Erhard Bremer (Philipps-University Marburg, Germany).

### Molecular cloning and nucleic acid procedures

Standard procedures were followed for generation of knock-out constructs and complementation plasmids (38). Plasmids are based on the plasmids pJET2.1 (Thermo Fisher Scientific), petef-GFP-Ala6-MMXN (39), and pNEBUC (20). pNEBUC is an ARS plasmid with carboxin resistance constructed for *U. maydis* but also suitable for *SrS* (Fig. S2A). Genomic DNAs of *U. maydis* and *S. reilianum f. sp. reilianum* cells were prepared according to an established protocol (40). Primer sequences are listed in Table S2, and plasmids are listed in Table S3. Plasmids were verified by sequencing. RNA was isolated as described using TRIzol (Invitrogen) and the Monarch Spin RNA Cleanup Kit (New England Biolabs) (41).

### qRT-PCR

RNA was prepared from strains grown 6 h in 2% glucose containing YNB-liquid media lacking nitrogen or in 2% glucose containing YNB-liquid media supplemented with 0.1% ammonium sulfate. A 25 mL culture with a starting OD_600_ = 0.2 was used for RNA preparation. qRT-PCR was performed with 20 ng RNA using the Luna Universal One-Step RT-qPCR Kit (New England Biolabs) on a Biorad CFX384 Touch Real-Time PCR Detection System. *gapdh* was used as the reference gene (42). The reaction conditions indicated by the manufacturer were applied. Reaction conditions were as follows: 10 min at 55°C, 1 min at 95°C followed by 40 cycles of 10 s at 95°C, 30 s at 60°C and a plate read step, and then the melt curve was performed at 55 to 95°C.

### Strain construction

Transformation of *U. maydis* and *S. reilianum f. sp. reilianum* was conducted as described (43). Constructs for gene replacement using geneticin (G-418, Sigma-Aldrich) resistance for selection in *S. reilianum f. sp. reilianum* were generated as described previously using an *Sfi*1-based cassette system (pMF1-G) and a PCR-based cloning strategy (20, 44). For complementation experiments in *U. maydis,* plasmids were linearized with SspI. Integration of plasmids into the *ip*-locus of *U. maydis* can produce carboxin-resistant strains (45). Integration of plasmids was verified by Southern blot analysis (38). For complementation experiments in *S. reilianum f. sp. reilianum,* the expression cassettes from the *U. maydis* complementation plasmids were amplified by PCR (T7, SP6) and cloned in the SmaI restriction site of pNEBUC, a plasmid that harbors an autonomously replicating sequence and enables carboxin resistance (Fig. S2A) (20). Purified ARS-plasmids (200 ng) were transformed into the corresponding protoplasts.

### Analysis of glycolipids

Extracellular glycolipids were extracted as previously described (10). Glycolipids (MELs and CLs) were separated by thin-layer chromatography (TLC) on silica plates, first with a solvent system consisting of chloroform-methanol-water (65:25:4, vol/vol/vol) for 5 min, followed by a second solvent system consisting of chloroform-methanol (9:1, vol/vol; 2 × 18 min) (46). The plates were dried, and sugar-containing compounds were visualized by application of a mixture of ethanol: sulfuric acid: p-anisaldehyde (18:1:1, vol/vol/vol), followed by heating at 150°C for 2 min (47).

### HPLC-MS(MS) analysis

High-performance liquid chromatography (HPLC) separation of the extracted MELs (50 µL) was performed with a 1260-HPLC system (Agilent) equipped with a EC 125/2 Nucleodur 100-3 C8 ec column (Macherey-Nagel). The gradient applied at a flow rate of 0.2 mL/min and a column temperature of 45°C was as follows (buffer A is water with 0.05% formic acid; buffer B is methanol with 0.045% formic acid): linear gradient from 60% buffer B to 95% buffer B within 30 min and then holding 95% buffer B for 10 min.

Online electrospray ionization MS and MS^2^ of the HPLC-separated compounds was done with an Orbitrap QExactive Plus mass spectrometer (Thermo Fisher Scientific). Electrospray ionization parameters were adapted to the flow rate and mass range. Accurate precursor ion masses (accuracy, 3 ppm), allowing the determination of the chemical formulas of the eluting compounds, were obtained by using the Orbitrap mass analyzer at a resolution of 140,000 within a mass range of 400–1,200 *m/z*. Data-dependent MS^2^-spectra were recorded with a resolution of 17,500, whereby five consecutive MS^2^-scans were measured with a stepped normalized collision energy of each 25, 30, and 35, following a precursor ion scan. The accurate masses in combination with MS^2^ experiments were sufficient to identify the acylation pattern of the compounds. Data were analyzed using the software Xcalibur (Thermo Fisher Scientific).

### Determination of surface tension

Surface tension was determined using the K20 force tensiometer (Krüss, Germany). Fifteen milliliters of deionized water was mixed with increasing amounts of MELs. The surface tension was measured using a Wilhelmi plate, and the critical micelle concentration (CMC) was determined. The CMC is defined as the concentration of surfactants above which micelles form, and all additional surfactants added to the system will form micelles (48). Experiments were at least repeated three times with different MEL preparations.

### Emulsification

The potential of MELs to emulsify oil and fatty acids was analyzed according to the protocol of Fukuoka et al. (22) Briefly, 4 mL distilled water was mixed thoroughly for 1 min with 12,5 µL of MELs (10 µg/µL in methanol) and either 500 µL corn oil (Tegut, Germany), 15 W-40 (Liqui Moly, Germany) or octanoic acid (caprylic acid) (Sigma-Aldrich, Germany). The mixture was incubated for 18 h at room temperature, and the turbidity was measured photometrically at 620 nm. Methanol was used as the control.

### Antibacterial activity

The antibacterial effects of MELs were measured in growth assays using a microplate reader (CLARIOstar^Plus^, BMG Labtech). A volume of 100 µL of bacterial cells of OD_600_ = 0.1 was grown at 23°C in LB medium (Roth, Germany) in the presence of MELs. Growth was recorded every 30 min for 20 h. The graphs represent the OD_600_ when the control cells [1] reached the stationary phase (approx. 13 h). LD50 was determined by V[mL] x c[mg/mL] / 0.1 mL.

### BLAST searches

Database searches were done using the blastp and blastn platforms of the National Center for Biotechnology Information (NCBI; https://www.ncbi.nlm.nih.gov).

### Alignments

Alignments were generated with the help of Clustal Omega (https://www.ebi.ac.uk/Tools/msa/clustalo/).

## Data Availability

NCBI accession numbers are as follows: *S. reilianum* f. sp*. reilianum* Mat1 (SRS1_14176; SJX63356), Mmf1 (SRS1_14177; SJX63357), Mac1 (SRS1_14178; SJX63358), Emt1 (SRS1_14179; SJX63359), Mac2 (SRS1_21039; SJX63360), Mat2 (SRS1_12505; SJX61519), and Mac3 (SRS1_12506; SJX61520); *S. reilianum* SRZ2 Mat1 (Sr14176; CBQ73519), Mmf1 (Sr14177; CBQ73520), Mac1 (Sr14178; CBQ73521), and Emt1 (Sr14179; CBQ73522); *U. maydis* (*Mycosarcoma maydis*) Mat1 (UMAG_03114; XP_011389465), Mmf1 (UMAG_03115; XP_011389466), Mac1 (UMAG_03116; XP_011389467), Emt1 (UMAG_03117; XP_011389468), Mac2 (UMAG_10636; XP_011389530), Mat2 (UMAG_01436; XP_011387305), and Mac3 (UMAG_01438; XP_011387307); *S. scitamineum* Mat2 (SPSC_03737; CDR88151) and Mac3 (SPSC_03738; CDR88152); *S. graminicola* Mat2 (Ex895_001892; XP_029741346) and Mac3 (Ex895_001891; XP_029741345); and *S. exsertum* BioProject PRJEB50360 (NCBI).

## SUPPLEMENTAL MATERIAL

Supplemental material mbio.00899-25-s0001.pdf Fig. S1-S9; Tables S1-S3.

ASM does not own the copyrights to Supplemental Material that may be linked to, or accessed through, an article. The authors have granted ASM a non-exclusive, world-wide license to publish the Supplemental Material files. Please contact the corresponding author directly for reuse.
