# Supplementary material for: Two gene clusters are required for mannosylerythritol lipid biosynthesis in Sporisorium reilianum
Source: mBio. 2025 Aug 18;16(9):e00899-25. doi: 10.1128/mbio.00899-25 (PMC12421966; doi:10.1128/mbio.00899-25)
Supplement: Supplemental material — Fig. S1-S9; Tables S1-S3. [file mbio.00899-25-s0001.pdf]

Supplementals for Tiefenbacher et al:

Two gene clusters are required for Mannosylerythritol lipid biosynthesis in *Sporisorium reilianum*

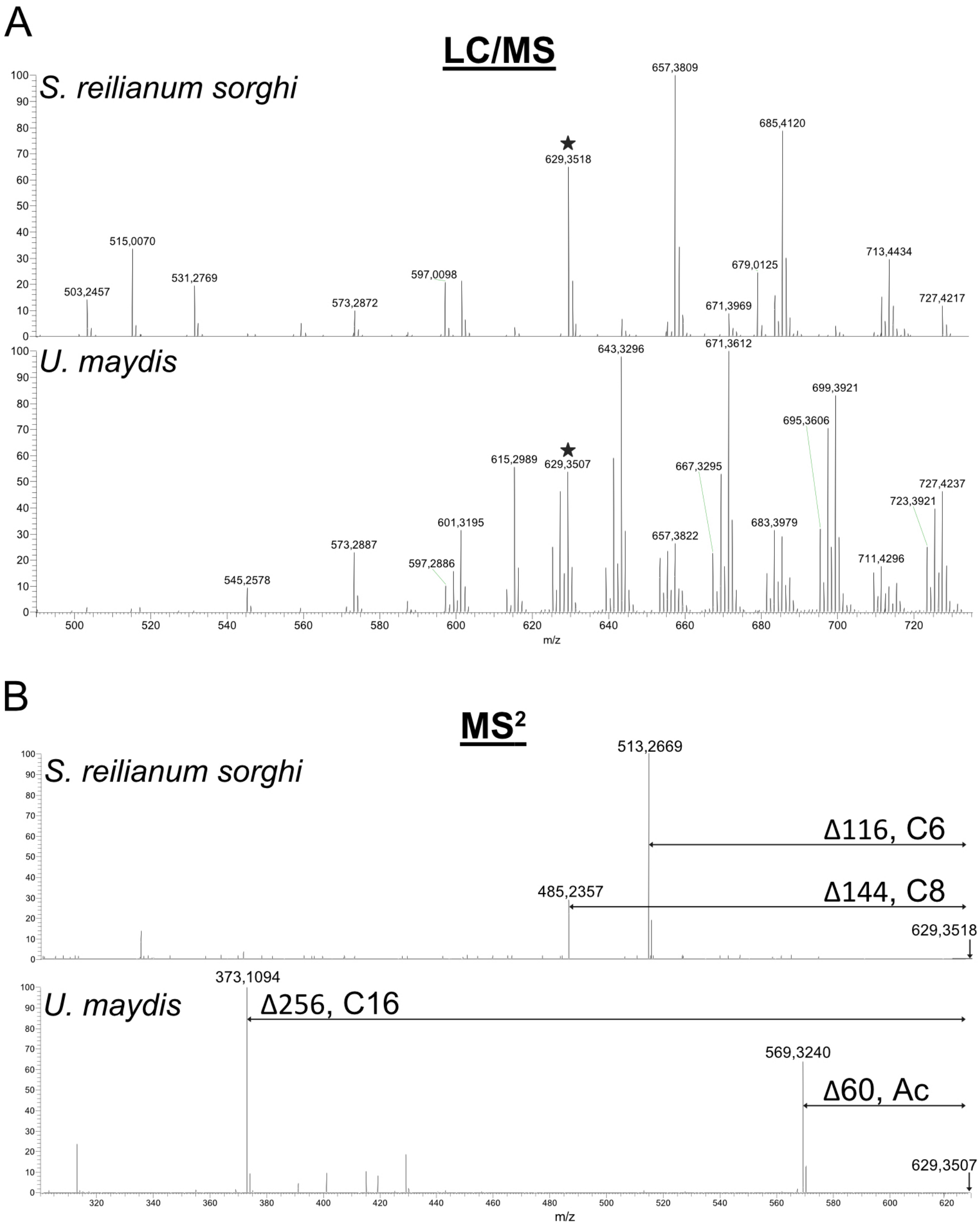

Figure S1: Mass spectrometry of MELs from *SrS* and *Um*

A: Total ion count spectra of MELs from *SrS* and *Um* analyzed by LCMS. B: Mass fragmentation (MS^2) of m/z=629,35 for *SrS* and *Um*. Ac=acetate, C6=hexanoic acid (C_6), C8=octanoic acid (C_8), C16=palmitic acid (C_{16}).

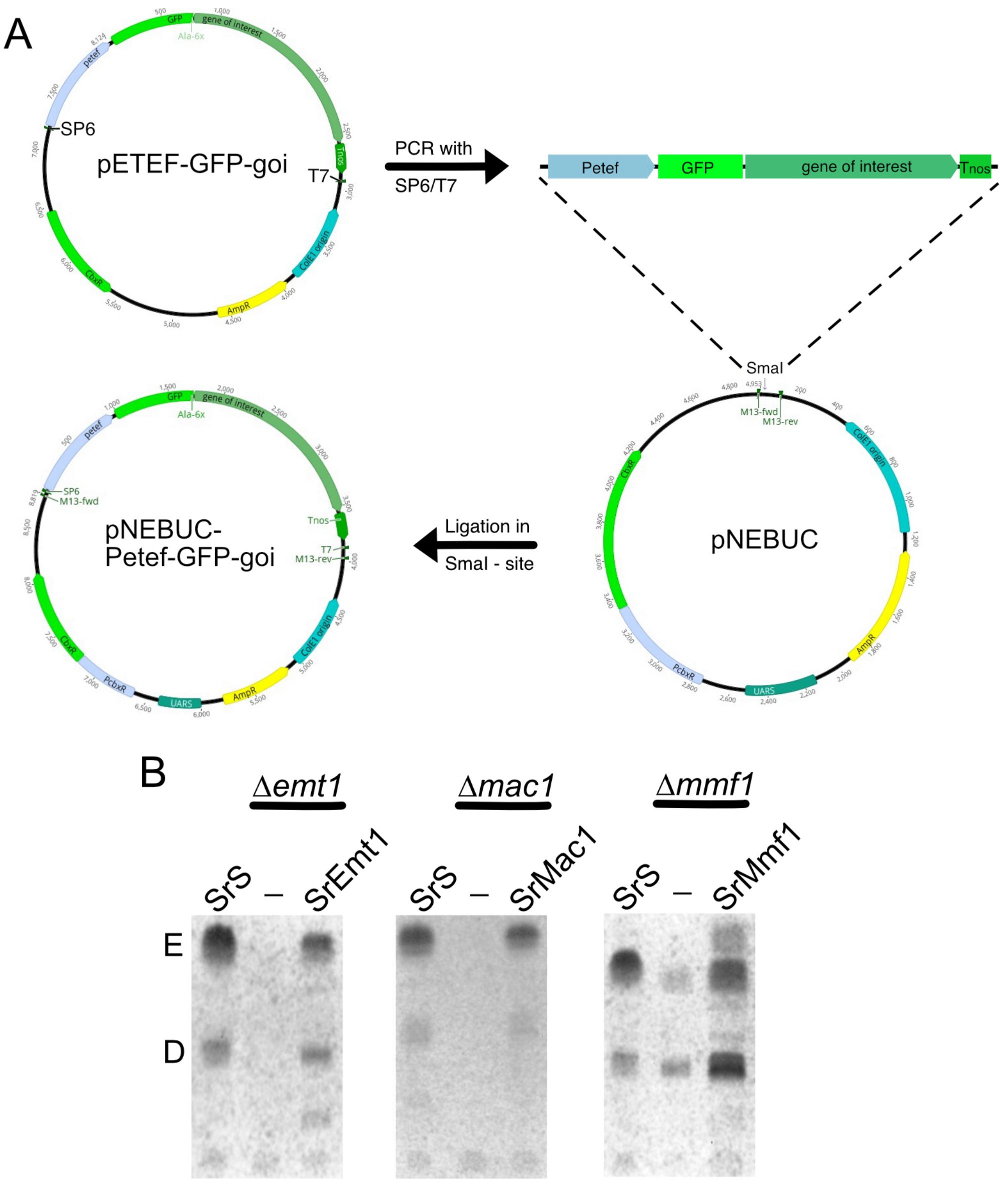

Figure S2: Complementation of the *SrS* deletion mutants with the corresponding MEL genes.

A: Cloning strategy to obtain expression constructs for *SrS* on the ARS plasmid pNEBUC. B: TLC of MELs synthesized by the rescued *SrS* deletion strains.

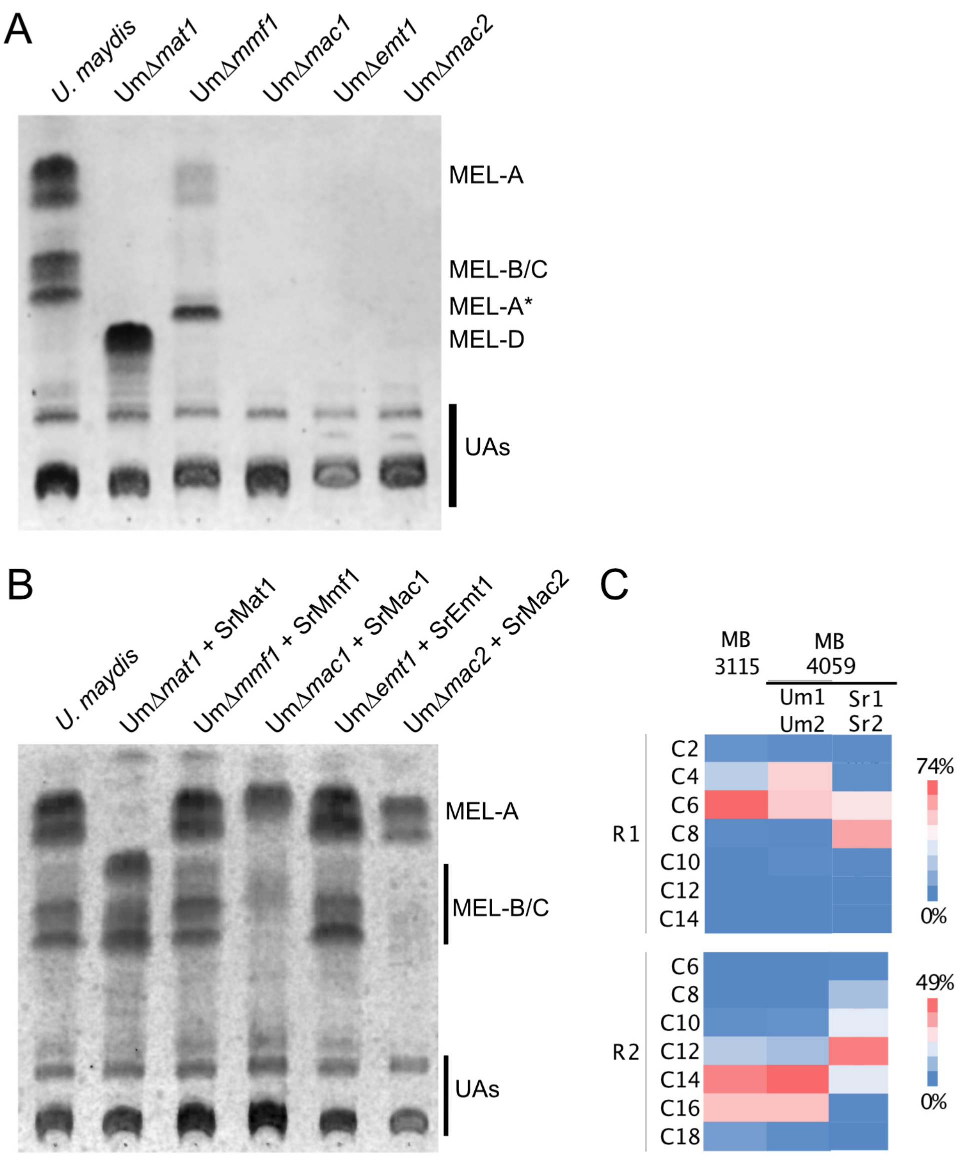

Figure S3: MELs from *U. maydis* wt and deletion strains

A: MELs from *U. maydis* wt strain MB215 and from the MEL gene cluster deletion mutants as indicated. MEL-A* are mono-acylated MEL variants (15) B: Heterologous complementation of the *Um* deletion mutants with the corresponding MEL genes of *SrS*. C: Heat maps showing the incorporation pattern of fatty acids at R1 and R2 for MB3115 expressing UmMac1 and UmMac2 (21) and for a strain expressing SrMac1 and SrMac2.

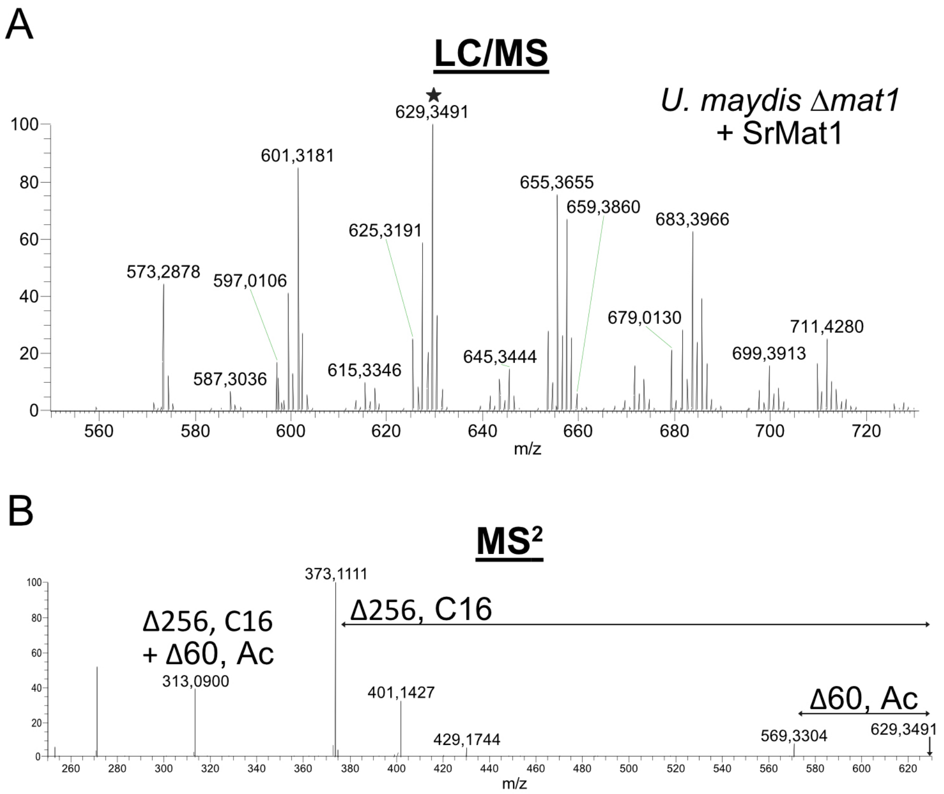

Figure S4: LCMS analysis of MELs from *Um*Δ*mat1* complemented with SrMat1
A: Total ion count spectra of MELs from the indicated strain analyzed by LCMS. B: Mass fragmentation (MS^2) of m/z=629,35. Ac= acetate, C16= C_{16} fatty acid (Palmitic acid).

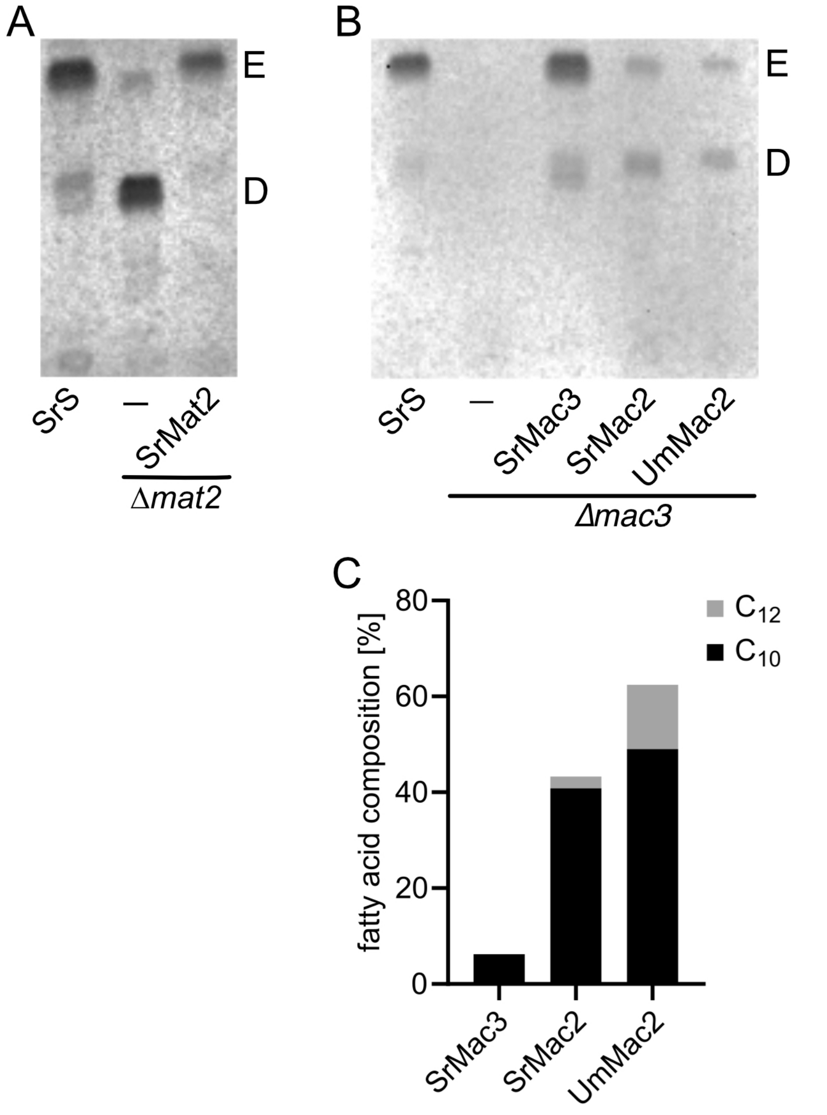

Figure S5: Analysis of MELs from rescued *SrS* deletion strains
A: Complementation of *SrS*Δ*mat2*: TLC of MELs extracted from the indicated strains B: Complementation of *SrS*Δ*mac3*: TLC of MELs extracted from the indicated strains. C: Percentage of C_{10} and C_{12} fatty acids measured in MS^2 fragmentation of MELs from B.

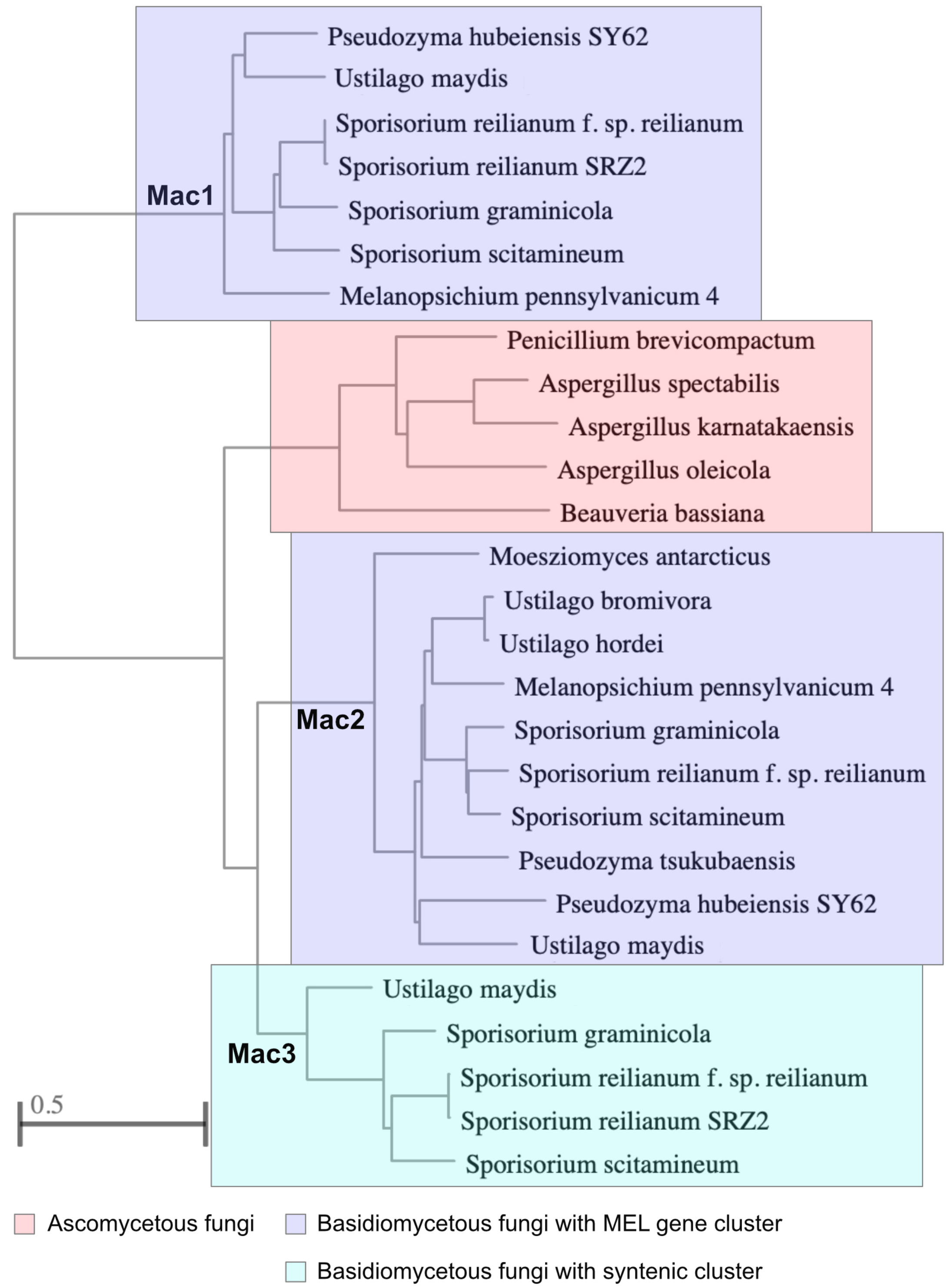

Figure S6: Phylogenetic analysis of SrMac3
BLASTp from NCBI (https://blast.ncbi.nlm.nih.gov) was used to identify relatives of SrS_Mac3. The accession numbers for the proteins used to build the phylogenetic tree using NCBI are:
Mac3: XP_011387307.1 (*Ustilago maydis*), XP_029741345.1 (*Sporisorium graminicola*), SJX61520.1 (*Sporisorium reilianum f. sp. reilianum*), CBQ70845.1 (*Sporisorium reilianum SRZ2*), CDR88152.1 (*Sporisorium scitamineum*)
Mac2: CDU26158.1 (*Sporisorium scitamineum*), SAM82151.1 (*Ustilago bromivora*), XP_041410023.1 (*Ustilago hordei*), CD153945.1 (*Melanopsichium pennsylvanicum 4*), BBE07955.1 (*Pseudozyma tsukubaensis*), XP_011389530.1 (*Ustilago maydis*),

XP_029740444.1 (*Sporisorium graminicola*), XP_012190144.1 (*Pseudozyma hubeiensis SY62*)
Ascomycetous fungi: KAL4872506.1 (*Aspergillus spectabilis*), XP 056807367.1 (*Penicillium brevicompactum*), XP_073577512.1 (*Aspergillus karnatakaensis*), KAL4943079.1 (*Aspergillus oleicola*), KAF1731931.1 (*Beauveria bassiana*)
Mac1: XP_012190147.1 (*Pseudozyma hubeiensis SY62*), CD153947.1 (*Melanopsichium pennsylvanicum 4*), XP_011389467.1 (*Ustilago maydis*), SJX63358.1 (*Sporisorium reilianum f. sp. reilianum*), CBQ73521.1 (*Sporisorium reilianum SRZ2*), CDU26160.1 (*Sporisorium scitamineum*), XP_029740446.1 (*Sporisorium graminicola*)

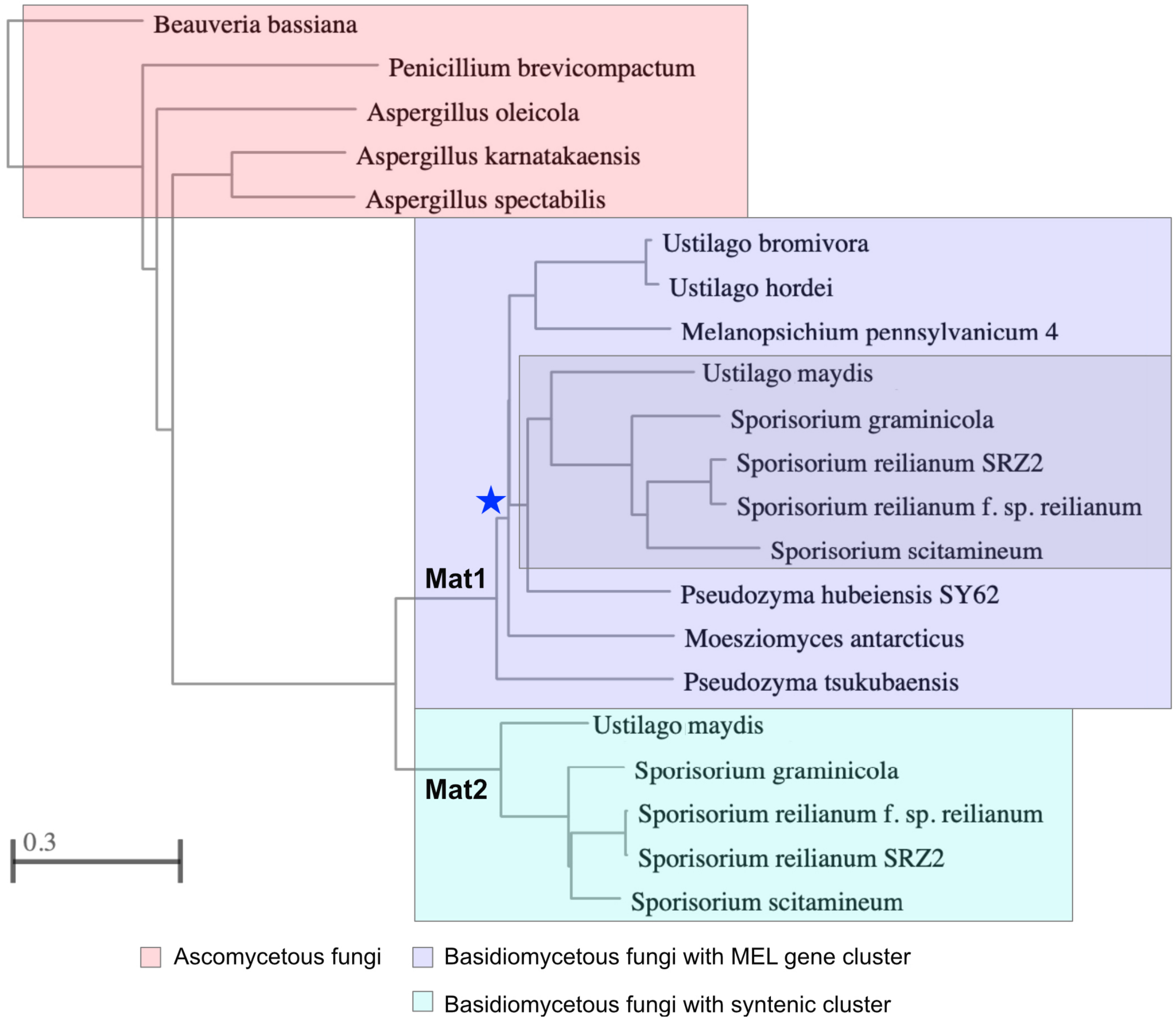

Figure S7: Phylogenetic analysis of SrMat2

BLASTp from NCBI (https://blast.ncbi.nlm.nih.gov) was used to identify relatives of SrS_Mat2. The accession numbers for the proteins used to build the phylogenetic tree using NCBI are:

Mat2: XP_012190147.1 (*Pseudozyma hubeiensis SY62*), CD153947.1 (*Melanopsichium pennsylvanicum 4*), XP_011389467.1 (*Ustilago maydis*), SJX63358.1 (*Sporisorium reilianum f. sp. reilianum*), CBQ73521.1 (*Sporisorium reilianum SRZ2*), CDU26160.1 (*Sporisorium scitamineum*), XP_029740446.1 (*Sporisorium graminicola*)

Mat1: XP_011387305.1 (*Ustilago maydis*), XP_029741346.1 (*Sporisorium graminicola*), SJX61519.1 (*Sporisorium reilianum f. sp. reilianum*), CBQ70844.1 (*Sporisorium reilianum SRZ2*), CDR88151.1 (*Sporisorium scitamineum*)

Mac2: CDU26162.1 (*Sporisorium scitamineum*), SAM82157.1 (*Ustilago bromivora*), XP_041410027.1 (*Ustilago hordei*), CD153949.1 (*Melanopsichium pennsylvanicum 4*), BBE15484.1 (*Pseudozyma tsukubaensis*), XP_011389465.1 (*Ustilago maydis*), XP_029740448.1 (*Sporisorium graminicola*), XP_012190149.1 (*Pseudozyma hubeiensis SY62*)

Ascomycetous fungi: KAL4872507.1 (*Aspergillus spectabilis*), KAJ5339644.1 (*Penicillium brevicompactum*), XP_073577513.1 (*Aspergillus karnatakaensis*), KAL4943077.1 (*Aspergillus oleicola*), PMB69703.1 (*Beauveria bassiana*)

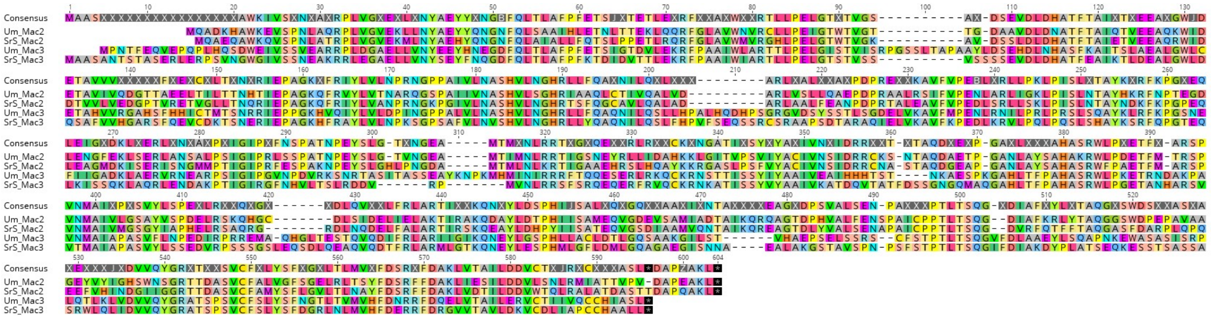

Figure S8: Sequence alignment of Um_Mac2, Um_Mac3, SrS_Mac2 and SrS_Mac3
Protein sequences (see figure S6) were aligned using the pairwise alignment tool of Geneious^{TM}.

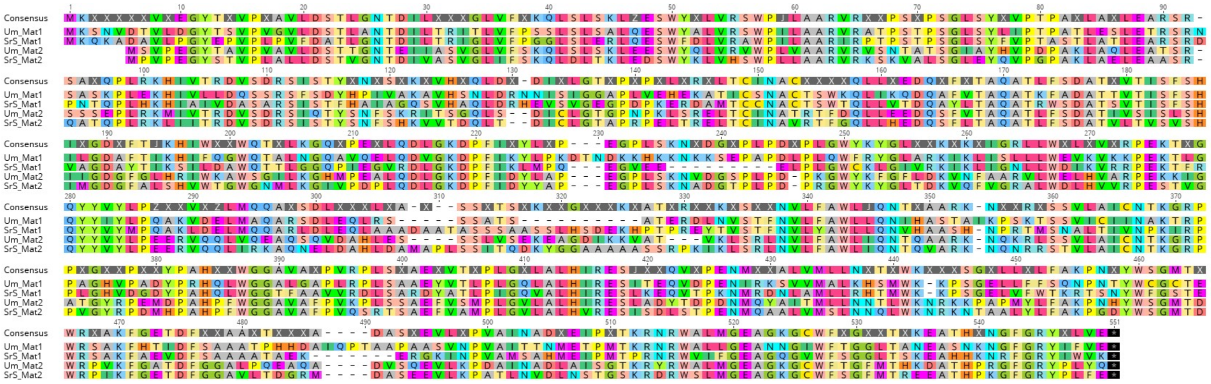

Figure S9: Sequence alignment of Um_Mat1, Um_Mat2, SrS_Mat1 and SrS_Mat2
Protein sequences (see figure S7) were aligned using the pairwise alignment tool of Geneious^{TM}.

Supplementary Table 1. Strains used in this study

Strain	Reference	DNA transformed	Integration	Progenitor strain
Ustilago maydis MB215	(Hewald et al., 2005)			
MB215 Δ*mat1* (MB3115)	(Hewald et al., 2006)			
MB215 Δ*rua1*	(Teichmann et al., 2008)			
MB215 Δ*mac1* Δ*mac2* Δ*mat1* (MB4059)	(Becker et al., 2021)			
MB215 Δ*mat1*+ Petef-GFP-SrMat1		pETEF-GFP-SrMat1	*ip*	MB215 Δ*mat1*
MB215 Δ*mat1*+ Petef-GFP-SrMat2		pETEF-GFP-SrMat2	*ip*	MB215 Δ*mat1*
MB215 mCherry-SKL	(Freitag et al., 2014)			
MB215 mCherry-SKL + Petef-GFP-SrMat2		pETEF-GFP-SrMat2	*ip*	MB215 Δ*mat1*
MB215 Δ*mmf1*	(Becker et al., 2022)			
MB215 Δ*mmf1*+ Petef-GFP-SrMmf1		pETEF-GFP-SrMmf1	*ip*	MB215 Δ*mmf1*
MB215 Δ*mac1*	(Hewald et al., 2006)			
MB215 Δ*mac1* + Petef-GFP-SrMac1		pETEF-GFP-SMmac1	*ip*	MB215 Δ*mac1*
MB215 Δ*emt1*	(Hewald et al., 2005)			
MB215 Δ*emt1* + Petef-GFP-SrEmt1		pETEF-GFP-SrEmt1	*ip*	MB215 Δ*emt1*
MB215 Δ*mac2*	(Hewald et al., 2006)			
MB215 Δ*mac2* + Petef-GFP-SrMac2		pETEF-GFP-SrMac2	*ip*	MB215 Δ*mac2*
MB215 Δ*mac2* + Petef-GFP-SrMac3		pETEF-GFP- SrSmac3	*ip*	MB215 Δ*mac2*
MB215 Δ*mac1* Δ*mac2* Δ*mat1* + Pmac1:GFP-SrSmac1 Pmmf1:mCherry-SrSmac2		pTB1-SrMac1-SrMac2	*ip*	MB215 Δ*mac1* Δ*mac2* Δ*mat1* (MB4059)
Sporisorium reilianum f. sp. reilianum (*SRS3* (H2)-8)	(Zuther et al., 2012)			
Sporisorium reilianum f. sp. zeae (*SRZCX112*)	(Schirawski et al., 2005)			
SRS Δ*mat1*		LF-gentR-RF (SrSmat1)	*mat1*	
SRS Δ*mmf1*		LF-gentR-RF (SrSmmf1)	*mmf1*	
SRS Δ*mmf1*+ Petef:GFP-SrMmf1		pNEBUC-Petef:GFP-SrMmf1		SRS Δ*mmf1*
SRS Δ*mac1*		LF-gentR-RF (SrSmac1)	*mac1*	
SRS Δ*mac1*+ Petef:GFP-SrMac1		pNEBUC-Petef:GFP-SrMac1		SRS Δ*mac1*

SRS Δ*emt1*	LF-gentR-RF (SrSemt1)	*emt1*	
SRS Δ*emt1*+ Petef:GFP-SrEmt1	pNEBUC-Petef:GFP-SrEmt1		SRS Δ*emt1*
SRS Δ*mac2*	LF-gentR-RF (SrSmac2)	*mac2*	
SRS Δ*mac3*	LF-gentR-RF (SrSmac3)	*mac3*	
SRS Δ*mac3*+ Petef:GFP-SrMac3	pNEBUC-Petef:GFP-SrMac3		SRS Δ*mac3*
SRS Δ*mac3*+ Petef:GFP-SrMac2	pNEBUC-Petef:GFP-SrMac2		SRS Δ*mac3*
SRS Δ*mac3*+ Petef:GFP-UmMac2	pNEBUC-Petef:GFP-UmMac2		SRS Δ*mac3*

Supplemental Table S2. Primers used in this study

Primer	used for	sequence
MM184	SrSmat1 LF 5‘	GTCGACCGGCGCTACGCTGGGCG
MM185	SrSmat1 LF 3‘ + SfiI	tga*ggcctgagtggcc*GCTTGTACGCTGTGAGCAGCACTC
MM008	SrSmat1 RF 5‘ + SfiI	ctc*ggccatctaggcc*CCAACCCATGGTGAATGGCAG
MM009	SrSmat1 RF 3‘ + SspI	ga*aatatt*GATCCCGATCTACTTCCAGTCG
MM187	SrSmmf1 LF 5‘	GTCTTTCAACCACCGGCCAACAC
MM186	SrSmmf1 LF 3‘ + SfiI	ttc*ggccatctaggcc*GCTGTTCCGGCAAGCGTCTTTGG
MM655	SrSmmf1 RF 3’	GGTGCAAGCTCGGGATCGTTCG
MM656	SrSmmf1 RF 5’ + SfiI	tga*ggcctgagtggcc*TGTCTCCCGTCTCTTTCGCTG
BS075	SrSmac1 LF 5‘	TTGCTTGTCACCGATCGAACACC
BS076	SrSmac1 LF 3‘ + SfiI	ata*ggcctgagtggcc*GGTGGCGAAGAGGCAGAGGGAAC
BS077	SrSmac1 RF 5‘ + SfiI	ggt*ggccatctaggcc*GCACGCTCATCCGCATTCGTCTC
BS078	SrSmac1 RF 3‘	ATTAAAGACACGCGACGCAGCTTC
BS079	SrSmac2 LF 5‘	GCACTGACGGTGACGTCTCGAGC
BS080	SrSmac2 LF 3‘ + SfiI	ata*ggcctgagtggcc*CTTGATGAAAGAAGGGTGCGCCTAC
BS081	SrSmac2 RF 5‘ + SfiI	ggt*ggccatctaggcc*GTCTCTTTTTGCTTCACTCTTGTC
BS082	SrSmac2 RF 3‘	ATTGATTTTGTAGTTGACCATCTTG
BS177	SrSmac3 LF 5‘	GCTCCAAAGGCGCCTCTTCCTG
BS178	SrSmac3 LF 3‘ + SfiI	ata*ggcctgagtggcc*GTTGGCTGATGCGGCCATGTTG
BS179	SrSmac3 RF 5‘ + SfiI	ggt*ggccatctaggcc*TCACAAGCGTCATCGTGATCTG
BS180	SrSmac3 RF 3‘	CAGCACCATCACGATGCCGGTC
BS181	gentR-fwd	GGCATGCGCGCCTTGAGCCTGG
BS182	gentR-rev	GATTGAACAAGATGGATTGCAC
BS214	SrSmat2 LF 5‘	ATCAAAGACCAAGGCTCGAGAG
BS215	SrSmat2 LF 3‘ + SfiI	cta*ggcctgagtggcc*GTCGATGACTGGCGCTCAGTTG
BS216	SrSmat2 RF 5‘ + SfiI	gat*ggccatctaggcc*CAGCATTCGTGGCGGGTGTTTC
BS217	SrSmat2 RF 3‘	ATCAAAGCTGTGAAACGACGCTG
BS222	SrSemt1 LF 5‘	ATCATGCCCACGGTTATCGCTC
BS223	SrSemt1 LF 3‘ + SfiI	cta*ggcctgagtggcc*GATGAGCTGATGTGGAGAGAAG
BS224	SrSemt1 RF 5‘ + SfiI	gat*ggccatctaggcc*CATGCTTCACAGTCAGTCACC
BS225	SrSemt1 RF 3‘	ATCTGTCTCTCGACGCCATGCG
MI449	SrSmat1 5‘ + EcoRV	atat*gatatc*tc**ATG**AAGCAAAAGGCAGACGCG
MI450	SrSmat1 3‘ + NotI	atat*gcggccgc*CTACTTGACCCAGATGTACC
MI016	SrSmmf1 5‘ + HpaI	ctaggatcc*tggccacc***ATG**ACGGGCGATAAGCGAGCATCG
MI018	SrSmmf1 3‘ + NotI	cta*gcggccgc*TCACGGCATTGTCGACGGCACCTCGG
BS198	SrSmac1 5‘ + SnaBI	gac*tacgta*cc**ATG**ATCAACAACGCGCTCCG
MI452	SrSmac1 3‘ + NotI	atat*gcggccgc*CTAGAGACGAGCAGACACC
ML975	SrSemt1 5‘ + MluI	cag*acgcgt*c**ATG**AAGGTCGCACTTCTCGCC
ML976	SrSemt1 3‘ + NotI	gta*gcggccgc*CTACTCGATGTTGGCGACGGTAG
BS147	SrSmac2 5‘ + SnaBI	gtc*tacgta*cc**ATG**CAGGCCGAACAAGCGTGG
BS148	SrSmac2 3‘ + NotI	gat*gcggccgc*CTAAAGCTTGGCTTGGGGAGCG
BS202	SrSmac3 5‘ + SnaBI	gac*tacgta*cc**ATG**GCCGCATCAGCCAACACTTC
ML798	SrSmac3 3‘ + NotI	cat*gcggccgc*CTAAAGCAAGGCAGCGTGGCAG
BS219	SrSmat2 5‘ + SnaBI	gac*tacgta*cc**ATG**CCAGTCCCCGAAGGATAC
BS220	SrSmat2 3‘ + NotI	cat*gcggccgc*TCATTCGAACAGTGGATATCGAC
BS149	Srei_qPCR_emt1fwd_3	AAGGACCTGTACGCACGAAG
BS150	Srei_qPCR_emt1rev_3	AACGTGATCTGGTGAGGCTG
BS153	Srei_qPCR_mat1fwd_3	TGCGACAAGTGTCACGATCA

BS154	Srei_qPCR_mat1rev_3	TCCCACAACAGGTTGCCAAT
BS155	Srei_qPCR_mac1fwd_3	GACTCGATCTACACGCCCAG
BS156	Srei_qPCR_mac1rev_3	TCCATCTTGTCCGCGATGAG
BS157	Srei_qPCR_mac2fwd_1	AATACTCGCTCGGCCATCTG
BS158	Srei_qPCR_mac2rev_1	CGATGCACGCGTAGACAAAG
BS159	Srei_qPCR_gapdh_fwd	GGATTTCATCGGCAACTCAC
BS160	Srei_qPCR_gapdh_rei	TACCACGAGACGAGCTTGAC
BS161	Srei_qPCR_mac3fwd_2	GCGCAAGTTCAAGACACGTT
BS162	Srei_qPCR_mac3rev_2	AGCTTCCGCGTTGTTACTGA
BS163	Srei_qPCR_mmf1fwd_5	CAGCACCTCGTCAAGGACAT
BS164	Srei_qPCR_mmf1rev_5	CAGCTCCTTGATCGCAAACG
BS233	Srei_qPCR_mat2fwd_3	GAACGTTTGGCCAGCTTCTG
BS234	Srei_qPCR_mat2rev_3	GATATGGCTGACCGAGACGG
SP6		ATTTAGGTGACACTATAG
T7		TAATACGACTCACTATAGGG

Supplementary Table S3

Plasmids constructed for this study.

Plasmid	used for	cloning procedure
pETEF-GFP-SrMat1	Complementation of UmΔ*mat1* (Hewald et al., 2006)	PCR product using Primers MI449 and MI450 was digested with EcoRV and NotI and cloned in MscI/NotI digested pETEF-GFP-Ala6-MMXN (Böhmer et al., 2008)
pETEF-GFP-SrMmf1	Complementation of UmΔ*mmf1*(Becker et al., 2022)	PCR product using Primers MI016 and MI018 was digested with HpaI and NotI and cloned in MscI/NotI digested pETEF-GFP-Ala6-MMXN (Böhmer et al., 2008)
pETEF-GFP-SrMac1	Complementation of UmΔ*mac1* (Hewald et al., 2006)	PCR product using Primers BS198 and MI452 was digested with SnaBI and NotI and cloned in MscI/NotI digested pETEF-GFP-Ala6-MMXN (Böhmer et al., 2008)
pETEF-GFP-SrEmt1	Complementation of UmΔ*emt1*(Hewald et al., 2005)	PCR product using Primers ML975 and ML976 was digested with MluI and NotI and cloned in MluI/NotI digested pETEF-GFP-Ala6-MMXN (Böhmer et al., 2008)
pETEF-GFP-SrMac2	Complementation of UmΔ*mac2* (Hewald et al., 2006)	PCR product using Primers BS147 and BS148 was digested with SnaBI and NotI and cloned in MscI/NotI digested pETEF-GFP-Ala6-MMXN (Böhmer et al., 2008)
pETEF-GFP-SrMac3	Complementation of UmΔ*mac2* (Hewald et al., 2006)	PCR product using Primers BS202 and ML798 was digested with SnaBI and NotI and cloned in MscI/NotI digested pETEF-GFP-Ala6-MMXN (Böhmer et al., 2008)
pETEF-GFP-SrMat2	Complementation of UmΔ*mat1* (Hewald et al., 2006)	PCR product using Primers BS219 and BS220 was digested with SnaBI and NotI and cloned in MscI/NotI digested pETEF-GFP-Ala6-MMXN (Böhmer et al., 2008)
pNEBUC-Petef:GFP-SrMat1	Complementation of SrSΔ*mat2*	PCR product using Primers SP6 and T7 on Plasmid pETEF-GFP-SrMat1 was cloned in SmaI digested pNEBUC (Brachmann et al., 2004)
pNEBUC-Petef:GFP-SrMmf1	Complementation of SrSΔ*mmf1*	PCR product using Primers SP6 and T7 on Plasmid pETEF-GFP-SrMmf1 was cloned in SmaI digested pNEBUC (Brachmann et al., 2004)
pNEBUC-Petef:GFP-SrMac1	Complementation of SrSΔ*mac1*	PCR product using Primers SP6 and T7 on Plasmid pETEF-GFP-SrMac1 was cloned in SmaI digested pNEBUC (Brachmann et al., 2004)

pNEBUC-Petef:GFP-SrEmt1	Complementation of SrSΔ*emt1*	PCR product using Primers SP6 and T7 on Plasmid pETEF-GFP-SrEmt1 was cloned in SmaI digested pNEBUC (Brachmann et al., 2004)
pNEBUC-Petef:GFP-SrMac2	Complementation of SrSΔ*ma3*	PCR product using Primers SP6 and T7 on Plasmid pETEF-GFP-SrMac2 was cloned in SmaI digested pNEBUC (Brachmann et al., 2004)
pNEBUC-Petef:GFP-SrMac3	Complementation of SrSΔ*mac3*	PCR product using Primers SP6 and T7 on Plasmid pETEF-GFP-SrMac3 was cloned in SmaI digested pNEBUC (Brachmann et al., 2004)
pNEBUC-Petef:GFP-SrMat2	Complementation of SrSΔ*mat2*	PCR product using Primers SP6 and T7 on Plasmid pETEF-GFP-SrMat2 was cloned in SmaI digested pNEBUC (Brachmann et al., 2004)
pNEBUC-Petef:GFP-UmMac2	Complementation of SrSΔ*mac3*	PCR product using Primers SP6 and T7 on Plasmid pETEF-GFP-UmMac2 (Freitag et al., 2014) was cloned in SmaI digested pNEBUC (Brachmann et al., 2004)
pJET1.2-koSrSmat1-gentR	Deletion of SrSΔ*mat1* by homologous recombination	1000 bp left flank (LF) and 1000 bp right flank (RF) were amplified by PCR on gDNA from SRS using the primer pairs MM184/185 and MM008/009, respectively. LF was cloned in pJet1.2. RF was added to the PvuII restriction site in the correct orientation. The geneticin resistance cassette (pMF1G, Brachmann et al., 2004) was cloned in the SfiI-sites of LF and RF. The entire ko-cassette was PCR amplified using the Primers MM184 and MM009.
pJET1.2-koSrSmmf1-gentR	Deletion of SrSΔ*mmf1* by homologous recombination	1000 bp left flank (LF) and 1000 bp right flank (RF) were amplified by PCR on gDNA from SRS using the primer pairs MM186/187 and MM655/656, respectively. LF was cloned in pJet1.2. RF was added to the PvuII restriction site in the correct orientation. The geneticin resistance cassette (pMF1G, Brachmann et al., 2004) was cloned in the SfiI-sites of LF and RF. The entire ko-cassette was PCR amplified using the Primers MM186 and MM656.
pJET1.2-koSrSmac1-gentR	Deletion of SrSΔ*mac1* by homologous recombination	1000 bp left flank (LF) and 1000 bp right flank (RF) were amplified by PCR on gDNA from SRS using the primer pairs BS075/076 and BS077/078, respectively. LF was cloned in pJet1.2. RF was added to the PvuII restriction site in the correct orientation. The geneticin resistance cassette (pMF1G, Brachmann et al., 2004) was cloned in the SfiI-sites of LF and RF. The entire ko-cassette was PCR amplified using the Primers BS075 and BS076.
pJET1.2-koSrSemt1-gentR	Deletion of SrSΔe*mt1* by homologous recombination	1000 bp left flank (LF) and 1000 bp right flank (RF) were amplified by PCR on gDNA from SRS using the primer pairs BS222/223 and BS224/225, respectively. LF was cloned in pJet1.2. RF was added to the PvuII restriction site in the correct orientation. The geneticin resistance cassette (pMF1G, Brachmann et al., 2004) was cloned in the SfiI-sites of LF and RF. The entire ko-cassette was PCR amplified using the Primers BS222 and BS225.
pJET1.2-koSrSmac2-gentR	Deletion of SrSΔ*mac2* by homologous recombination	1000 bp left flank (LF) and 1000 bp right flank (RF) were amplified by PCR on gDNA from SRS using the primer pairs BS079/080 and BS081/082, respectively. LF was cloned in pJet1.2. RF was added to the PvuII restriction site in the correct orientation. The geneticin resistance cassette (pMF1G, Brachmann et al., 2004) was cloned in the SfiI-sites of LF and RF. The entire ko-cassette was PCR amplified using the Primers BS079 and BS082.
pJET1.2-koSrSmac3-gentR	Deletion of SrSΔ*mac3* by homologous recombination	1000 bp left flank (LF) and 1000 bp right flank (RF) were amplified by PCR on gDNA from SRS using the primer pairs BS177/178 and BS179/180, respectively. LF was cloned in pJet1.2. RF was added to the PvuII restriction site in the correct orientation. The geneticin resistance cassette (pMF1G, Brachmann et al., 2004) was cloned in the SfiI-sites of LF and RF. The entire ko-cassette was PCR amplified using the Primers MBS177 and BS180.
pJET1.2-koSrSmat2-gentR	Deletion of SrSΔ*mat2* by homologous recombination	1000 bp left flank (LF) and 1000 bp right flank (RF) were amplified by PCR on gDNA from SRS using the primer pairs BS214/215 and BS216/217, respectively. LF was cloned in pJet1.2. RF was added to the PvuII restriction site in the correct orientation. The geneticin resistance cassette (pMF1G, Brachmann et al., 2004) was cloned in the SfiI-sites of LF and RF. The entire ko-cassette was PCR amplified using the Primers BS214 and BS217.
